# Neuropsychiatric Inventory domains cluster into neuropsychiatric syndromes in Alzheimer's disease: A systematic review and meta‐analysis

**DOI:** 10.1002/brb3.2734

**Published:** 2022-08-08

**Authors:** Shaun Kuan Wei Hiu, Theophile Bigirumurame, Patience Kunonga, Andrew Bryant, Manjunadh Pillai

**Affiliations:** ^1^ Population Health Sciences Institute, Newcastle University Newcastle University UK; ^2^ Campus for Ageing and Vitality, Northumberland Tyne and Wear, Newcastle upon Tyne Hospitals Newcastle upon Tyne UK

**Keywords:** Alzheimer's disease, meta‐analysis, neuropsychiatric symptoms, systematic review

## Abstract

**Background**: Studies of patients with Alzheimer's disease (AD) have observed that neuropsychiatric symptoms (NPS) tend to co‐occur as neuropsychiatric syndromes and have generally shown mixed results regarding the number and composition of syndromes. We systematically reviewed how neuropsychiatric syndromes in AD have been defined and compared the different published definitions in a pooled sample of AD patients using meta‐analytic structural equation modeling (MASEM).

**Methods**: Studies examining the factor structure of the Neuropsychiatric Inventory (NPI) and published from 1994 to 2021 were included. We contacted the corresponding authors of eligible studies for correlation coefficients between NPI items. We pooled correlations under a random effects MASEM model and fitted and compared measurement models from published studies to identify a best‐fitting model.

**Results**: Twenty‐five studies were included in the systematic review, and correlations were obtained from seven studies for MASEM. For the NPI‐10 (seven studies, *n* = 5185), a five‐factor structure was found to have a good fit to the data. For the NPI‐12 (four studies, *n* = 2397), we were unable to identify a factor structure that displayed a good model fit.

**Conclusion**: This systematic review and meta‐analysis contribute to the development of a theoretical model of neuropsychiatric syndromes in AD and reveals the barriers that accompany MASEM methodology.

## 1 INTRODUCTION

Neuropsychiatric symptoms (NPS) are a prevalent feature in patients with Alzheimer's disease (AD) and span a wide range of behavioral and psychological disturbances (Zhao et al., 2016). NPS has been shown to be associated with faster progression to severe dementia and death and poorer quality of life (Peters et al., 2015; Shin et al., 2005). As such, the evidence of their adverse consequences motivates the continued understanding of NPS. The Neuropsychiatric Inventory (NPI) is widely used to measure NPS and the ability to quantify owing to its symptom coverage frequency and severity of NPS. Two versions of the NPI are available: the 10‐item version covers delusions, hallucinations, agitation/aggression, depression, anxiety, euphoria, apathy, disinhibition, irritability, and aberrant motor behavior, while the 12‐item version additionally covers night‐time behavior disturbances and appetite and eating abnormalities (Cummings, 1997; Cummings et al., 1994).

One focus of research has been the development of theoretical models of neuropsychiatric syndromes in AD with the eventual goal of a unified theoretical model. Studies have consistently observed that certain NPS have a tendency to co‐occur with others, leading to efforts to systematically investigate “clusters” or “groupings” of symptoms termed neuropsychiatric syndromes. Sound theoretical models of neuropsychiatric syndromes have important benefits for guiding future research and treatment development and necessitate the integration of results from studies investigating the valid measurement of syndromes, plausible neurobiological explanations, and neuroimaging and biomarkers (Geda et al., 2013). They may offer hypotheses on etiological explanations for why certain symptoms tend to co‐occur as syndromes and the mechanisms linking neuropsychiatric syndromes to AD symptomology (Geda et al., 2013). Knowledge of the underlying neurobiology and mechanisms may then contribute to the development of therapies and the refinement of research diagnostic criteria to identify homogeneous patient populations to enrol in pharmacological and non‐pharmacological trials (Cummings, 2021). These models would also lead to consistent definitions of neuropsychiatric syndromes as outcomes, thus enabling the comparability of clinical trial results targeting syndromes and facilitate meta‐analyses of these trials.

Numerous analytic techniques, such as factor analysis and principal component analysis (PCA), have been used to identify neuropsychiatric syndromes (Aalten et al., 2007; Kang et al., 2010). A previous systematic review of neuropsychiatric syndromes in AD highlighted a large degree of variation regarding which syndrome each NPS loaded onto and additionally reported that the number of syndromes reported ranged from one to seven—with a slight majority of studies of the NPI observing a four‐syndrome structure (Canevelli et al., 2013). The review reported that despite the large variation in the number and makeup of syndromes, there was evidence that certain pairs of NPS tended to systematically co‐occur together such as delusions and hallucinations, irritability and agitation, depression and anxiety, euphoria and disinhibition.

We contributed to the advancement of the understanding of neuropsychiatric syndromes by investigating the factor structure of the NPI in the AD population. We first conducted a systematic review where we described how neuropsychiatric syndromes have been defined in patients with AD. We then compared the different factor structures in a meta‐analysis of the NPI that pools data across studies identified from the systematic review using the meta‐analytic structural equation modeling (MASEM) methodology, which offers a way to pool effect sizes (correlation coefficients between NPI scores) across studies and obtain robust evidence for the measurement model that offers the best representation of neuropsychiatric syndromes (Cheung, 2014). An additional objective of our review was to report on the feasibility of conducting a meta‐analysis when the required effect sizes were expected to be largely absent from publications. Our systematic review extended previous work (Canevelli et al., 2013) first as an update on published factor structures of neuropsychiatric syndromes. An update was essential to ensure we had up‐to‐date coverage of the published factor structures and that our meta‐analysis results were not biased due to the omission of a potentially reliable structure. Second, our review provided a risk of bias assessment for all included studies using the COnsensus‐based Standards for the selection of health Measurement INstruments (COSMIN) checklist (Mokkink et al., 2018). The inclusion of the COSMIN assessment allowed us to assess the quality and trustworthiness of the available evidence regarding the grouping of NPI domains into syndromes. Last, a limitation of the prior systematic review was that conclusions were solely based on the observed proportion of studies reporting various patterns of symptom‐to‐syndrome relationships. Our meta‐analysis overcomes this by providing direct comparisons of the different factor structures to identify the best‐fitting one using established model fit criteria.

## 2 MATERIALS AND METHODS

### 2.1 Search strategy

This review was registered on PROSPERO (ID: CRD42020211038). We searched for articles published between January 1, 1994 (the year the NPI was first published) and December 31, 2020, through the following databases: Pubmed; PsycINFO; Web of science; SCOPUS; MEDLINE via Ovid; and EMBASE via Ovid. The following keywords were used: “factor analysis” or “factor structure” or “latent structure” or “common factor” or “principal component” or dimension or psychometric or cluster and neuropsychiatric and inventory or symptoms or syndrome or syndromal or subsyndrome or subsyndromal or npi, and Alzheimer. A search update was carried out on December 27, 2021, to cover articles published in 2021. Gray literature was sought through the reference sections of published review articles on NPS in AD. All references were managed by EndNote. Screening for eligibility was conducted by two reviewers using Rayyan (SKWH and TB).

### 2.2 Selection criteria

The selection criteria were: original research published between 1994 and 2021; conducted on a sample of patients diagnosed with probable or possible AD using an established clinical criteria such as the (i) Diagnostic and Statistical Manual of Mental Disorders (DSM), for example, DSM‐III, DSM‐IV, DSM‐V, (ii) National Institute of Neurological and Communicative Disorders and Stroke/Alzheimer's Disease and Related Disorders Association (NINCDS‐ADRDA), (iii) revisions to the NINCDS‐ADRDA by the National Institute on Aging and the Alzheimer's Association workgroup (McKhann et al., 2011), or (iv) International Statistical Classification of Diseases and Related Health Problems (ICD), for example, ICD‐9, ICD‐10; use of the 10‐ or 12‐item NPI; performed PCA, exploratory factor analysis (EFA), confirmatory factor analysis (CFA), or latent class analysis using the NPI; and published in English. If multiple studies originated from the same participant pool, they were added to the systematic review, but the correlation coefficients from the study with the larger sample size were prioritized for the meta‐analysis. Studies using variants of the NPI (e.g., nursing homes) were also eligible. Studies that only used the caregiver distress component of the NPI or the NPI‐Questionnaire were excluded. All disagreements regarding study eligibility between authors were discussed until a consensus was reached.

### 2.3 Data extraction

Data (such as participant characteristics, country of study, study design, diagnostic criteria, version of the NPI used, and results) from the studies were extracted by one author (SKWH) using a standardized template. The quality and accuracy of the data were checked by a co‐author (PK). If the NPI inter‐item correlation matrix was not reported in a publication or supplemental material, the corresponding author was contacted via email. If no response was received after 2 weeks, a follow‐up email was sent, and authors were given an additional 2 weeks to respond. If the study had a longitudinal design, we requested the correlation matrix from the baseline visit data.

### 2.4 Risk of bias assessment

We adapted the COSMIN checklist to assess the risk of bias (Mokkink et al., 2018). As our objectives were mostly concerned with the structural validity of the NPI, we only based our evaluations on the internal structure criterion. The risk of bias assessment for all studies was carried out by one author (SKWH), and a random 20% of the studies was checked by a co‐author (PK).

### 2.5 Data analysis

We summarized the study sample characteristics, study design features, and results in tabular form. To describe how NPS co‐occurred with one another, we reported a co‐occurrence matrix (Shafer, 2005) where each cell describes the number of studies in which a particular pair of NPI items had their highest factor loadings on the same factor (for PCA and EFA) or were purposefully loaded onto the same factor (for CFA). If a loading matrix was not reported, we used the factor solution reported in text. For studies that explored multiple factor solutions, we selected the solution that the authors presented as their definitive solution.

We used MASEM for the meta‐analysis, particularly the two‐stage structural equation modeling (TSSEM) approach using the *metaSEM* package (Cheung, 2014, 2015; Cheung & Chan, 2005). The TSSEM methodology has useful applications in health research to synthesize information from results produced from confirmatory factor analysis (CFA), moderations and mediation analyses, and structural equation models to understand relationships between variables and, importantly, test theoretical models (Cheung & Hong, 2017). For the purposes of our meta‐analysis, it may be convenient to consider it as a type of “meta‐analytic confirmatory factor analysis” (Norton et al., 2013).

The primary analysis of the NPI‐10 (Cummings et al., 1994) involved pooling the inter‐item (Pearson's product‐moment) correlation coefficients, organized in matrix form, from studies using the 10‐ and 12‐item versions (excluding night‐time behavioral disturbances and appetite and eating abnormalities). A secondary analysis of the NPI‐12 (Cummings, 1997) was conducted on studies that used the NPI‐12 and involved the inter‐item correlations of all 12 items.

In stage one of TSSEM, we pooled study correlation matrices together under a random effects model. This was decided a priori because we did not assume a common population correlation matrix across all studies. Rather, it assumes that there is between‐study variation in population correlation matrices by treating studies as random samples from a larger population of possible studies. As such, random effects models allow for inferences beyond the studies being analyzed. To assess the homogeneity of effect sizes, the *Q*‐test and *I* ^2^ statistic for each of item–item correlation coefficients were reported (Cheung, 2014); a statistically significant *Q*‐test suggests that the effect sizes are not homogeneous across studies and a higher *I* ^2^ values indicate higher degrees of heterogeneity in that particular item–item correlation coefficient. The between‐study heterogeneity τ^2^ of an effect size was fixed at zero if its estimate reached the lower bound of 1e‐10 during pooling.

In stage two, we fitted various measurement models to the pooled correlation matrix and compared model fit indices: model χ^2^ statistic; comparative fit index (CFI), Tucker–Lewis index (TLI), root mean square error of approximation (RMSEA), standardized root mean square residual (SRMR), Akaike information criterion, and Bayesian information criterion. The criteria for good model fit are: SRMR < 0.08; RMSEA < 0.06 and CFI and the TLI > 0.95 (Hu & Bentler, 1999). We reported the estimated standardized coefficients and likelihood‐based 95% confidence intervals (CI) for all paths and residual variances. For the NPI‐10, the measurement models compared included three studies selected a priori (Garre‐Olmo et al., 2010; Spalletta et al., 2010; Vilalta‐Franch et al., 2010). For the NPI‐12, the measurement models compared included three other studies selected a priori (Aalten et al., 2007; Hollingworth et al., 2006; Mirakhur et al., 2004). Knowledge of the studies was obtained from a published review and they were pre‐selected owing to their “large” sample sizes (defined as *n* ≥ 300), suggesting a degree of reliability of the results (Canevelli et al., 2013). We also included measurement models from other large samples or CFA studies identified during the systematic review.

If the inter–item correlation matrix could not be obtained, an approximated correlation matrix may be computed using the information from the factor loadings table in studies using EFA and CFA. For CFA models or EFA with maximum likelihood estimation, model fit indices were assessed with the SRMR, RMSEA, CFI, and TLI criteria (Hu & Bentler, 1999) to determine how well the parameter estimates can closely reproduce the sample correlation matrix (see Supplemental Material 3 for application). For EFA with principal axis factoring, a conservative cut‐off of >80% total variance explained by the factors was chosen. We also reported the results of a sensitivity analysis that excluded the approximated correlation matrices.

## 3 RESULTS

### 3.1 Description of studies in the systematic review

**FIGURE 1:**
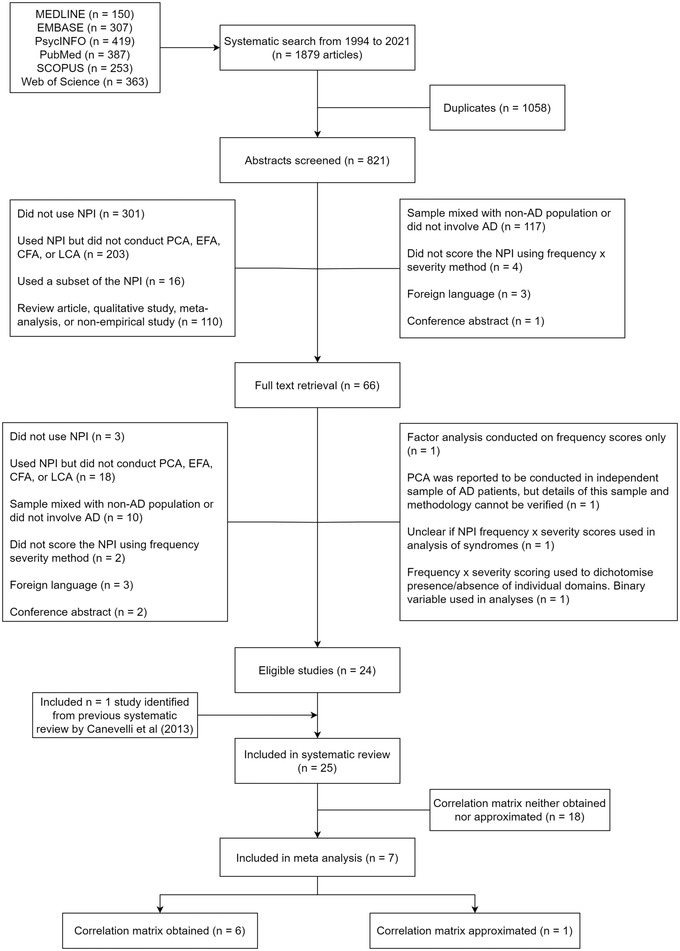
Preferred reporting items for systematic reviews and meta‐analyses flow chart. Abbreviations: AD, Alzheimer's disease; CFA, confirmatory factor analysis; EFA, exploratory factor analysis; NPI, Neuropsychiatric Inventory; PCA, principal component analysis

The results of our systematic search are summarized in Figure 1, according to the preferred reporting items for systematic reviews and meta‐analyses (PRISMA) guidelines (Page et al., 2021). In total, 25 studies were included in the systematic review (Aalten et al., 2007; Archer et al., 2007; Chen et al., 2012; Connors et al., 2018; Cummings et al., 2006; Dennehy et al., 2013; Frisoni et al., 1999; Garre‐Olmo et al., 2010; Gauthier et al., 2005; Germain et al., 2009; Hollingworth et al., 2006; Hwang et al., 2017; Kang et al., 2010; Kazui et al., 2016; Kim et al., 2021; Matsui et al., 2006; Mirakhur et al., 2004; Nagata et al., 2016; Poletti et al., 2013; Proitsi et al., 2011; Scassellati et al., 2020; Spalletta et al., 2010; Starr & Lonie, 2007; Vilalta‐Franch et al., 2010; Wang et al., 2012). The characteristics of these studies are summarized in Supplemental Material 1. The COSMIN risk of bias ratings is available in Supplemental Material 2. Overall, the risk of bias for structural validity was low as studies generally had adequate sample sizes and used appropriate methodology. There is a high risk of bias overall regarding the internal consistency of each syndrome as measures of reliability such as Cronbach's alpha are seldom reported. There is also a reasonable level of risk of bias overall concerning measurement invariance. Only one study provided information on differential item functioning of NPI items using multiple indicators and multiple causes modeling (Proitsi et al., 2011). Regarding longitudinal invariance (Putnick & Bornstein, 2016), only three studies had longitudinal data (Connors et al., 2018; Garre‐Olmo et al., 2010; Vilalta‐Franch et al., 2010); all studies performed PCA on the data at each follow‐up visit to identify the factor structure of the NPI‐10 at each measurement point, but only one study conducted a multi‐group CFA to assess for invariance of factor loadings over time (Connors et al., 2018).

The study sample sizes ranged from 96 (Chen et al., 2012) to 2188 (Aalten et al., 2007) AD patients. The mean study age ranged from 72 to 84 years. The percentage of female participants ranged from 50.3% (Connors et al., 2018) to 78.1% (Chen et al., 2012), and the mean mini‐mental state examination (MMSE) ranged from approximately 8 (Gauthier et al., 2005) to 23 (Hwang et al., 2017) points. The studies represented a wide range of geographic regions including Asia (*n* = 7, including Japan, Mainland China, South Korea, and Taiwan [Republic of China]), Australia (*n* = 1), Europe (*n* = 13, including England, Greece, Italy, Northern Ireland, Republic of Ireland, Scotland, Spain, and Wales), and North America (*n* = 4 from the United States).

The majority of studies used the NPI‐12(16/25; 64%). Most studies (18/25; 72%) used PCA as their primary method of analysis to draw conclusions on the factor structure of the NPI (Aalten et al., 2007; Archer et al., 2007; Chen et al., 2012; Cummings et al., 2006; Frisoni et al., 1999; Gauthier et al., 2005; Germain et al., 2009; Hollingworth et al., 2006; Kazui et al., 2016; Kim et al., 2021; Matsui et al., 2006; Mirakhur et al., 2004; Nagata et al., 2016; Poletti et al., 2013; Scassellati et al., 2020; Spalletta et al., 2010; Vilalta‐Franch et al., 2010), followed by CFA (5/25; 20%; Connors et al., 2018; Dennehy et al., 2013; Garre‐Olmo et al., 2010; Kang et al., 2010; Proitsi et al., 2011), and EFA (2/25; 8%; Hwang et al., 2017; Wang et al., 2012). The number of factors identified ranged from three to five with most studies supporting a three‐factor (11/25; 44%) structure followed by a four‐factor (9/25; 36%) and five‐factor (5/25; 20%) structure. A small percentage of studies (4/25; 16%) yielded factor solutions that discarded certain NPS as they either fell below a loading threshold on a factor to be classified or were not included prior to analysis (Aalten et al., 2007; Dennehy et al., 2013; Kang et al., 2010; Nagata et al., 2016). Most often, euphoria was discarded (3/4; 75%) followed by aberrant motor behavior (2/4; 50%).

The co‐occurrence matrices for the NPI‐10 and NPI‐12 are presented in Tables 1 and 2, respectively. For the NPI‐10, the most frequently reported item pairs (pairs of NPI items whose largest loadings were on the same factor) were delusions and hallucinations; agitation and irritability; depression and anxiety; disinhibition and aberrant motor behavior; and euphoria and disinhibition. Much like the NPI‐10, the most frequently reported item pairs for the NPI‐12 were also delusions and hallucinations; agitation and irritability; depression and anxiety; and euphoria and disinhibition; but additionally included depression and apathy; apathy and appetite and eating abnormalities; disinhibition and irritability; and hallucinations and night‐time behavioral disturbances. Single‐item factors were uncommon and appeared in six studies (Chen et al., 2012; Cummings et al., 2006; Matsui et al., 2006; Poletti et al., 2013; Spalletta et al., 2010; Wang et al., 2012); these were limited to euphoria, apathy, and appetite and eating abnormalities.

**TABLE 1:** Co‐occurrence matrix for Neuropsychiatric Inventory (NPI)‐10

**NPI items**	**1**	**2**	**3**	**4**	**5**	**6**	**7**	**8**	**9**	**10**
1. Delusions	–	8 (89)	2 (22)	0	1 (11)	0	0	1 (11)	2 (22)	3 (33)
2. Hallucinations		–	2 (22)	0	1 (11)	0	0	1 (11)	2 (22)	3 (33)
3. Agitation			–	3 (33)	4 (44)	0	2	3 (33)	8 (89)	4 (44)
4. Depression				–	7 (78)	1 (11)	3 (33)	0	3 (33)	0
5. Anxiety					–	1 (11)	2 (22)	0	3 (33)	0
6. Euphoria						1 (11)	1 (11)	5 (56)	1 (11)	3 (33)
7. Apathy							1 (11)	4 (44)	2 (22)	4 (44)
8. Disinhibition								–	3 (33)	6 (67)
9. Irritability									–	4 (44)
10. Aberrant motor behavior										–

*Note*: Numbers on diagonal indicate that the number of studies where the NPI item was the only item on a factor. Numbers in parentheses are the item pairings as a percentage (%) of included NPI‐10 studies. If no loading matrix was available, in‐text information was used. If an NPS loaded onto multiple factors, we included the pairings for all scenarios. Studies with no information regarding which NPI items loaded onto which factor were not included in tabulation.

**TABLE 2:** Co‐occurrence matrix for NPI‐12

**NPI items**	**1**	**2**	**3**	**4**	**5**	**6**	**7**	**8**	**9**	**10**	**11**	**12**
1. Delusions	–	13 (81)	5 (31)	2 (13)	4 (25)	1 (6)	0	2 (13)	4 (25)	2 (13)	4 (25)	0
2. Hallucinations		–	2 (13)	1 (6)	1 (6)	2 (13)	1 (6)	2 (13)	1 (6)	3 (19)	7(44)	2 (13)
3. Agitation			–	4 (25)	7 (44)	1 (6)	3 (19)	7(44)	15 (94)	4 (25)	1 (6)	2 (13)
4. Depression				–	12 (75)	2 (13)	8 (50)	4 (25)	4 (25)	1 (6)	2 (13)	2 (13)
5. Anxiety					–	1 (6)	5 (31)	1 (6)	6 (38)	0	0	2 (13)
6. Euphoria						1 (6)	1 (6)	10 (63)	2 (13)	4 (25)	2 (13)	1 (6)
7. Apathy							–	3 (19)	2 (13)	5 (31)	6 (38)	9 (56)
8. Disinhibition								–	8 (50)	5 (31)	2 (13)	1 (6)
9. Irritability									–	3 (19)	1 (6)	2 (13)
10. Aberrant motor behavior										–	7(44)	5 (31)
11. Night‐time behavioral disturbances											–	7(44)
12. Appetite and eating abnormalities												3 (19)

*Note*: Numbers on diagonal indicate that the number of studies where the NPI item was the only item on a factor. Numbers in parentheses are the item pairings as a percentage (%) of included NPI‐12 studies. If no loading matrix was available, in‐text information was used. If an NPS loaded onto multiple factors, we included the pairings for all scenarios. Studies with no information regarding which NPI items loaded onto which factor were not included in tabulation.

The percentage of zeroes in NPI domain scores was reported in 9/25 (36%) studies (Archer et al., 2007; Chen et al., 2012; Frisoni et al., 1999; Garre‐Olmo et al., 2010; Gauthier et al., 2005; Hollingworth et al., 2006; Mirakhur et al., 2004; Poletti et al., 2013; Scassellati et al., 2020). The observed range of percentage of zeroes for each domain were as follows: delusions (50%−84%), hallucinations (61%−94%), agitation (33%−80%), depression (30%−60%), anxiety (46%–69%), euphoria (74%−97%), apathy (24%−58%), disinhibition (69%−85%), irritability (35%−63%), aberrant motor behavior (35%−81%), night‐time behavioral disturbances (44%−73%), and appetite and eating abnormalities (36%−74%). The implications of the zero scores are included in our discussion.

### 3.2 Description of studies in meta‐analysis

The response rate for the correlation matrices was 6/25 (24%; Connors et al., 2018; Garre‐Olmo et al., 2010; Germain et al., 2009; Kang et al., 2010; Nagata et al., 2016; Scassellati et al., 2020); five corresponding authors provided the matrix while one shared anonymized individual patient data. Of the 19 studies, five corresponding authors reported that they either did not have the data or no longer had access to the data, five emails were no longer in use (attempts at locating and contacting more recent email addresses did not yield responses), and the remainder did not respond to our data request. An approximate correlation matrix was also computed from one CFA study (Proitsi et al., 2011). The correlation matrices used in the meta‐analysis are presented in Supplemental Material 3.

Most studies (4/7; 57.1%) used the NPI‐12. The mean age of study samples ranged from 73.2 to 80.5 years, the percentage of female participants ranged from 50.3% to 70.9%, and the mean MMSE ranged from 11.1 to 21.1 points. The study samples spanned multiple geographic regions including Australia, England, Greece, Italy, Northern Ireland, the Republic of Ireland, South Korea, Spain, Wales, and the United States. Compared to the 18 studies that were not included in the meta‐analysis, the ranges of mean age of the study samples were comparable (70.9–84 years) and PCA was still the most frequently used method of analysis (83.3%). There was a slightly higher proportion of female participants in the non‐included studies based on the range (57%–74%) and a relatively lower range for the mean MMSE (7.7–20.3). There was also a greater representation of Asian countries in the non‐included studies including those conducted in Mainland China, Taiwan (Republic of China), and Japan; these regions were not represented in the meta‐analysis.

### 3.3 Factor structure of NPI‐10

**FIGURE 2:**
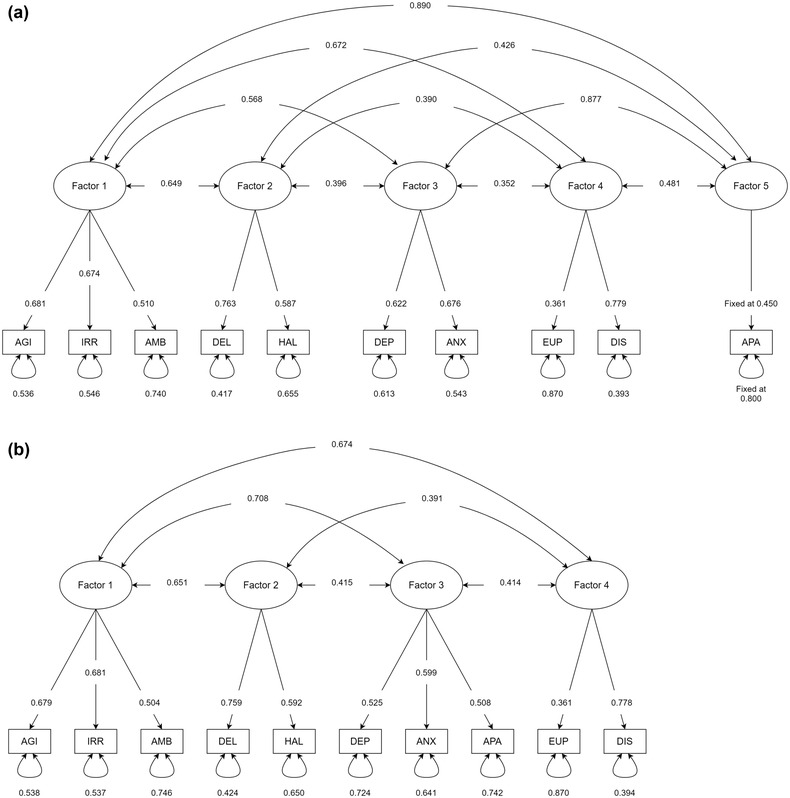
Diagram of the best‐fitting structure of the Neuropsychiatric Inventory (NPI)‐10. All figures are standardized coefficients. Bi‐directional arrows between factors indicate inter‐factor correlations. Bi‐directional arrows looping onto NPI items indicate error variances (proportion of variance of the NPI item that is unexplained by the factor). Unidirectional arrows from factors to NPI items indicate factor loadings. (a) (top): The original five‐factor measurement model in Spalletta et al. (b) (bottom): The modified four‐factor Spalletta et al.’s (2010) model is presented as Model C in Table 3. AGI, agitation; AMB, aberrant motor behavior; ANX, anxiety; APA, apathy; DEL, delusions; DEP, depression; DIS, disinhibition; EUP, euphoria; HAL, hallucinations; IRR, irritability

Seven correlation matrices (six obtained, one approximated) were included in the primary analysis and pooled under a random effects model and had a total sample size of 5185 AD patients (Supplemental Material 4a). The *Q*‐test for heterogeneity (*Q* = 942.87, degrees of freedom [df] = 270, *p* < .001) and *I* ^2^ values indicated that effect sizes were unlikely to be homogeneous across studies (Supplemental Material 4b), supporting the use of a random effects model. We compared seven measurement models from five studies using the NPI‐10 (Connors et al., 2018; Garre‐Olmo et al., 2010; Proitsi et al., 2011; Spalletta et al., 2010; Vilalta‐Franch et al., 2010; Table 3). We found that the five‐factor Spalletta et al. (2010) model (Figure 2a) had the best model fit across all indices and satisfied most of the criteria of good model fit (Hu & Bentler, 1999). A limitation was the presence of a single‐indicator factor, whereby apathy was the only NPI item on the fifth factor, which required the factor loading and error variance to be fixed during estimation (Brown, 2006). We assumed the latent factor explained a small proportion of the variance in apathy and fixed the error variance *e* = 0.80 and the factor loading at $\sqrt{1-e}\approx 0.45$ (Brown, 2006). We observed that as *e* approached 0 (and as factor 5 explained greater variation in apathy), the inter‐factor correlations with factor 5 became smaller. We explored four modifications of this measurement model that allowed the factor loading and error variance of apathy to be freely estimated and found an alternative four‐factor model (modified Spalletta et al., 2010, Model C in Table 3) that also met most of the criteria for good model fit (Figure 2b). Factor loadings, inter‐factor correlations, and error variances of the two measurement models are presented in Supplemental Material 4c and 4d. A sensitivity analysis with only the six observed correlation matrices (*n* = 3335 AD patients) maintained the same pattern of results and did not alter our conclusion (Supplemental Material 4e–4i).

**TABLE 3:** Comparison of model fit indices across measurement models for the NPI‐10

**Study and model**	**Factor 1**	**Factor 2**	**Factor 3**	**Factor 4**	**Factor 5**	**Model χ^2^**	**df**	**RMSEA [95% CI]**	**SRMR**	**TLI**	**CFI**	**AIC**	**BIC**
Garre‐Olmo et al. (2010) Model 1^a^	DEL HAL AMB	AGI DEP ANX APA IRR	EUP DIS	–	–	261.246	32	0.037 [0.033, 0.041]	0.058	0.873	0.910	197.246	−12.467
Garre‐Olmo et al. (2010) Model 2	DEL HAL	AGI DEP ANX APA IRR	EUP DIS AMB	–	–	274.025	32	0.038 [0.034, 0.042]	0.053	0.866	0.905	210.025	0.313
Garre‐Olmo et al. (2010) Model 3	DEL HAL	AGI DEP ANX IRR	EUP APA DIS AMB	–	–	304.419	32	0.041 [0.036, 0.045]	0.057	0.850	0.893	240.419	30.706
Proitsi et al. (2011)^b^	EUP APA AMB	DEL HAL	DEP ANX	AGI DIS IRR	–	312.495	29	0.043 [0.039, 0.048]	0.047	0.827	0.889	254.495	64.443
Vilalta‐Franch et al. (2010) follow‐up model	AGI DEP ANX IRR	DEL HAL APA	EUP DIS AMB	–	–	352.990	32	0.044 [0.04, 0.048]	0.066	0.823	0.874	288.990	79.277
Connors et al. (2018)^c^	DEL HAL	AGI DEP ANX IRR	APA DIS AMB	–	–	189.150	24	0.036 [0.032, 0.041]	0.054	0.884	0.923	141.150	−16.135
Spalletta et al. (2010)	AGI IRR AMB	DEL HAL	DEP ANX	EUP DIS	APA	113.606	26	0.026 [0.021, 0.030]	0.031	0.940	0.966	61.606	−108.786
Modified Spalletta et al. (2010) A	AGI APA IRR AMB	DEL HAL	DEP ANX	EUP DIS	–	171.903	29	0.031 [0.027, 0.035]	0.035	0.913	0.944	113.903	−76.150
Modified Spalletta et al. (2010) B	AGI IRR AMB	DEL HAL APA	DEP ANX	EUP DIS	–	227.828	29	0.036 [0.032, 0.041]	0.052	0.879	0.922	169.828	−20.224
Modified Spalletta et al. (2010) C	AGI IRR AMB	DEL HAL	DEP ANX APA	EUP DIS	–	142.368	29	0.028 [0.023, 0.032]	0.036	0.931	0.955	84.368	−105.684
Modified Spalletta et al. (2010) D	AGI IRR AMB	DEL HAL	DEP ANX	EUP APA DIS	–	258.115	29	0.039 [0.035, 0.044]	0.047	0.860	0.910	200.115	10.062

Abbreviations: AGI, agitation; AIC, Akaike information criterion; AMB, aberrant motor behavior; ANX, anxiety; APA, apathy; BIC, Bayesian information criterion; CFI, comparative fit index; df, degrees of freedom; DEL, delusions; DEP, depression; DIS, disinhibition; EUP, euphoria; HAL, hallucinations; IRR, irritability; RMSEA, root mean square error of approximation; SRMR, standardized root mean square residual; TLI, Tucker–Lewis index.

^a^ Same as the Vilalta‐Franch et al. (2010) baseline model.

^b^ Disinhibition loaded only on F3.

^c^ Measurement model does not have Euphoria.

### 3.4 Factor structure of NPI‐12

Four obtained correlation matrices were included in the primary analysis and pooled under a random effects model and had a total sample size of 2397 AD patients (Supplemental material 5a). The *Q*‐test (*Q* = 427.52, df = 198, *p* < .001) and *I* ^2^ indices both suggested that effect size estimates were unlikely to be homogeneous (Supplemental Material 5b). We compared five measurement models from five studies using the NPI‐12 (Aalten et al., 2007; Hollingworth et al., 2006; Kang et al., 2010; Mirakhur et al., 2004; Nagata et al., 2016). We did not find a model that satisfied most criteria for good model fit (Table 4), though the four‐factor Kang et al. (2010) model stood out as the relatively better fitting model (Supplemental Material 5c).

**TABLE 4:** Comparison of model fit indices across measurement models for the NPI‐12

**Study and model**	**Factor 1**	**Factor 2**	**Factor 3**	**Factor 4**	**Model χ^2^**	**df**	**RMSEA [95% CI]**	**SRMR**	**TLI**	**CFI**	**AIC**	**BIC**
Aalten et al. (2007)^a^	AGI DIS IRR AMB	DEL HAB NBD	DEP ANX	APA APP	231.264	38	0.046 [0.041, 0.052]	0.046	0.811	0.869	155.264	−64.451
Hollingworth et al. (2006)	EUP DIS AMB NBD APP	DEL HAL	DEP ANX APA	AGI IRR	312.032	48	0.048 [0.043, 0.053]	0.044	0.777	0.838	216.032	−61.503
Mirakhur et al. (2004)	AGI DEP ANX IRR	APA AMB NBD APP	DEL HAL	EUP DIS	317.148	48	0.048 [0.043, 0.054]	0.055	0.773	0.835	221.148	−56.387
Nagata et al. (2016)^b^	AGI IRR	APA APP	DEL HAL	DEP EUP DIS	185.765	21	0.057 [0.050, 0.065]	0.046	0.765	0.863	143.765	22.343
Kang et al. (2010)^c^	AGI DIS IRR	DEP ANX	DEL HAL	APA NBD APP	185.483	29	0.048 [0.041, 0.054]	0.041	0.821	0.885	127.483	−40.194

Abbreviations: AGI, agitation; AIC, Akaike information criterion; AMB, aberrant motor behavior; ANX, anxiety; APA, apathy; APP, appetite and eating abnormalities; BIC, Bayesian information criterion; CFI, comparative fit index; df, degrees of freedom; DEL, delusions; DEP, depression; DIS, disinhibition; EUP, euphoria; HAL, hallucinations; IRR, irritability; RMSEA, root mean square error of approximation; SRMR, standardized root mean square residual; TLI, Tucker–Lewis index.

^a^ Measurement model does not include Euphoria and is the same as the Dennehy et al. (2013) model.

^b^ Measurement model does not include anxiety, aberrant motor behavior, and night‐time behavioral disturbances.

^c^ Measurement model does not include euphoria and aberrant motor behavior.

## 4 DISCUSSION

In summary, this systematic review and meta‐analysis provided evidence of a biologically plausible measurement model of the NPI‐10 based on a large pooled sample of AD patients. However, evidence concerning the NPI‐12 was not sufficient to identify a suitable measurement model. From a clinical perspective, identifying syndromes by grouping together a number of manifestations that frequently co‐occur has advantages over a unitary concept of NPS due to the inherent heterogeneity and complex nature of the underlying major neurocognitive disorder. Due to the major influence of NPS on quality of life, morbidity, and long‐term prognosis, having an understanding of syndromes can help clinicians customize the management options and provide important clues to the etiopathology of dementias.

The findings from our meta‐analysis indicated that a five‐factor solution to the NPI‐10—agitation, irritability, and aberrant motor behavior (Factor 1); delusions and hallucinations (Factor 2); depression and anxiety (Factor 3); euphoria and disinhibition (Factor 4); and apathy (Factor 5)—was the best‐fitting model across a majority of model fit indices. Factor 1 encompassing agitation, irritability, and aberrant motor behavior has emerged in several studies under various names, indicating mostly frontal involvement and co‐existence of psychomotor features, and behavioral manifestation. There is evidence that this factor remains stable across a 31‐month period (Selbaek & Engedal, 2012). It would be useful to understand whether an early onset of these features indicates an anterior progression in AD or even an atypical variant. Factor 2 comprising delusions and hallucinations may represent the syndrome of “psychosis.” AD patients with psychotic symptoms have shown severe abnormalities in gray matter volume, cerebral blood flow, and metabolism in temporal, parietal and frontal cortices (Sweet et al., 2002) and cholinergic deficits (Tsang et al., 2006). Studies suggest a strong genetic influence on psychosis in AD, suggesting an important role for apolipoprotein E4 (APOE4) (Ismail et al., 2011) and other genes like *COMT* and *5HT2A* receptor polymorphism. Clinically, patients often develop secondary delusional beliefs in response to the persistent hallucinatory experience and treatment often improves both these symptoms, indirectly suggesting a common etiopathological origin. Factor 3 consisting of depression and anxiety is another commonly reported syndrome in AD. These NPS share common neuropathology including changes in neurotransmitters and abnormalities in the frontal‐limbic circuit and amygdala (Chen, Dang, & Zhang, 2021). Pharmacological treatment options are also similar in both these manifestations (Cummings et al., 2019). The NPS in Factor 4, euphoria and disinhibition, are among the least frequently reported manifestations in AD (Zhao et al., 2016). Current evidence suggest shared etiopathology, that is, frontal involvement especially reduction in the right frontal cortical thickness in patients with predominant disinhibition (Finger et al., 2017). These symptoms are highly debilitating for caregivers and early identification can help in the formulation of management plans accordingly. Apathy as a standalone factor is an important finding. Apathy is associated with severe cognitive deficits, significant caregiver burden, functional decline, and overall impact of the condition (Landes et al., 2001). Even though it is a frequent occurrence in dementia, the multidimensional nature of apathy—comprising behavioral, cognitive, and emotional symptoms—conveys challenges in terms of assessment, measurement and quantification, and treatment (Miller et al., 2021; Mortby et al., 2022). Recent diffusion imaging studies suggest an association of apathy with extensive white matter damage and dysfunction in the fronto‐subcortical cingulate pathways regardless of the sub‐type of dementia (Hollocks et al., 2015; Tay et al., 2019). Distinguishing between depression and apathy is an ongoing research challenge (Lanctot et al., 2017). From a clinical perspective, prominent apathy indicates a severe nature of illness independent of depression. Further research is needed to understand apathy as a standalone syndrome.

Evidence for a suitable factor structure of the NPI‐12 was weaker in comparison. None of the measurement models put forth met the majority of the criteria of good model fit. One surprising observation was the fact that the Aalten et al. (2007) model did not stand out as the better fitting model in comparison despite coming from the largest, and perhaps most cited, an empirical study identifying neuropsychiatric syndromes in AD to date. The fit indices for the Aalten et al. (2007) measurement model observed in our study were consistent with a study applying CFA (Dennehy et al., 2013), whereby criteria were met for RMSEA and SRMR but not TLI. Nevertheless, there was very low certainty evidence for the factor structure of the NPI‐12 owing to the small number of studies pooled, thus no strong assertions can be made.

The strength of our review was that we were able to pool effect size measures across studies to further our understanding of the factor structure of the NPI, thereby offering additional insights into neuropsychiatric syndromes in AD. The effect size data from Spalletta et al. (2010) were not included in our meta‐analysis, so the fact that their measurement model was the best fitting model of the NPI‐10 adds credibility to our results. Our systematic review findings were consistent with a previous review (Canevelli et al., 2013) regarding the choice of methodology (PCA most common), the number of factors (three and four were most common), frequency of single‐item factors (24% vs. 20%), and the extensive heterogeneity in the factor solutions derived. The major differences between our reviews are found in the item pairings. The authors pooled results across NPI versions and reported that the most frequent (≥ 60%) item pairs were delusions and hallucinations, irritability and agitation, depression and anxiety, and euphoria and disinhibition (Canevelli et al., 2013). However, when split by NPI versions, we noted differences between versions with regards to frequent pairings. While the delusions and hallucinations, irritability and agitation, and depression and anxiety pairs were > 60% for both versions of the NPI, the pairing of euphoria and disinhibition occurred in 63% of NPI‐12 studies but 56% of NPI‐10 studies. Furthermore, we observed that the aberrant motor behavior and disinhibition pairing occurred in 67% of NPI‐10 studies but only 31% of NPI‐12 studies. Lowering this threshold to 50% revealed that the apathy and depression pairing and irritability and disinhibition pairing occurred in 50% of NPI‐12 studies but both only occurred in 33% of NPI‐10 studies. This observation revealed potential variability in the pattern in which NPS is being grouped, depending on whether the correlations between NPI‐10 items and the night‐time behavioral disturbances and appetite and eating abnormalities domains are additional factors during analysis. These findings suggest that it may not necessarily be valid to group NPI‐10 domains into syndromes using factor solutions derived from the NPI‐12 and vice versa.

The MASEM methodology complemented the systematic review by recognizing that heterogeneity and allowed us to draw conclusions on the specific measurement model may best represent the data. This review conducted comprehensive searches of six bibliographic databases and gray literature and also followed procedures documented by the Cochrane Collaboration for conducting systematic reviews, the PRISMA guidelines for reporting (Page et al., 2021), and COSMIN for assessing the risk of bias (Mokkink et al., 2018). Our review was also able to report on the feasibility of conducting MASEM research in the study of neuropsychiatric syndromes.

A limitation of our review was the small number of studies being pooled. While this was not a problem for convergence, the number of studies may have contributed to an instance where the upper limit of the 95% CI for the residual variance of delusions in one measurement model could not be estimated. Furthermore, there is low certainty in our meta‐analysis results as our conclusions may be different had there been more available correlation matrices. This was an anticipated limitation of the data collection strategy given its reliance on the corresponding authors’ responses. It is plausible that publication bias plays a role such that investigators may selectively choose not to submit their results if their factor solution was not novel or had a statistically significant model χ^2^ fit statistic (suggesting lack of fit). At present, there is uncertainty over how to detect and adjust for publication bias in studies using MASEM, but it is hoped that future studies conducted at a time where such adjustment methods are widely accepted will be able to utilize our results. Another limitation due to the small number of studies was the inability to examine the presence of a higher‐order latent factor of “total/overall neuropsychiatric burden” that was pre‐specified in our protocol. The total score of the NPI tends to be used in analyses, even those of clinical trials (van den Elsen et al., 2015), but the structural validity of this construct has not been thoroughly investigated. We had fit a second‐order factor in which the latent factors were loaded onto this construct but encountered estimation issues such as negative residual variances (Heywood case) that were likely caused by insufficient data. NPI domain scores are observed to be right‐skewed and zero‐inflated—the latter of which may induce positive correlations between what should be independent (positive) discrete random variables. We identified that all NPI domains are affected by at least some degree of zero‐inflation but delusions, hallucinations, euphoria, and disinhibition were the most affected. A study that sought to analyze the NPI factor structure after adjusting for the zero‐inflation revealed that if zero‐inflation was unaccounted for then truly small and weak loadings were inflated and large loadings were suppressed (Hellton et al., 2021). Although it may be plausible that the loading estimates in our results are biased by the zero‐inflation inherent in NPI domain scores, there is uncertainty over whether the zero‐inflation had an effect (and the extent of that effect) on the model fit indices that were used as the criteria for establishing the best model. Statistical simulation studies of the TSSEM are warranted to determine if its weighted least squares estimation method is robust to ordinal indicator variables with zero inflation.

The results should not be taken as definitive to inform clinical practice but as a milestone in the pathway toward refining existing theoretical models of neuropsychiatric syndromes in AD (Geda et al., 2013) and the eventual development of a unified theoretical model. A recommendation for future MASEM investigations would be to carefully consider a trade‐off between data collection and resources. Our decision to restrict our inclusion criteria to studies investigating syndromes was a design decision as we wanted to systematically review those studies as well. Technically, if one wishes to investigate a factor structure, then a correlation matrix from any study that has a sample of clinically diagnosed AD participants with NPI data is fit for the purpose. This raises a logistical problem of an unfeasibly large number of studies to include. This revelation elevates the role that multi‐national collaborations have toward advancing syndrome research as they may possess the necessary resources and outreach to maximize data collection.

## AUTHOR CONTRIBUTIONS

Shaun Kuan Wei Hiu designed the review, collected, analyzed, interpreted the data, and provided critical input in writing the manuscript. Theophile Bigirumurame designed the review, provided expert statistical advice, collected and interpreted the data, and provided critical input in writing the manuscript. Patience Kunonga designed the review, collected and interpreted the data, and provided critical input in writing the manuscript. Andrew Bryant designed the review, interpreted the data, and provided critical input in writing the manuscript. Manjunadh Pillai designed the review, interpreted the data, and provided critical input in writing the manuscript.

## CONFLICT OF INTEREST

All authors declare no competing interests.

### PEER REVIEW

The peer review history for this article is available at: https://publons.com/publon/10.1002/brb3.2734.

## Supporting information

Supplemental Material 1: Study characteristics

Supplemental Material 2: COSMIN risk of bias ratings

**Supplemental Material 3**: The data presented below are copied verbatim from the email correspondences with study corresponding authors to preserve authenticity unless otherwise specified. Notes made by the study author (SKWH) are highlighted in yellow.

**Supplemental Material 4**: The information presented in this supplemental material concern the primary analysis of the NPI‐10 and its sensitivity analysis.

Supplemental Material 5: The information presented in this supplemental material concern the primary analysis of the NPI‐10 and its sensitivity analysis.

## Data Availability

The authors declare the data were collected from publicly available sources. Permission was sought from corresponding authors to analyze their effect size data and share them in a supplemental material. Data and code can be provided upon reasonable request.
