# Supplementary material for: Neuropsychiatric Inventory domains cluster into neuropsychiatric syndromes in Alzheimer's disease: A systematic review and meta‐analysis
Source: Brain Behav. 2022 Aug 8;12(9):e2734. doi: 10.1002/brb3.2734 (PMC9480932; doi:10.1002/brb3.2734)
Supplement: Supplementary file 1 — Supplemental Material 1: Study characteristics [file BRB3-12-e2734-s002.docx]

Supplemental material 1: Study characteristics

| **Study** | **N** | **Centres** | **Country** | **Design** | **Mean age** | **Females** | **Mean education** | **Mean MMSE** | **Recruitment source** | **Diagnostic criteria** | **NPI version** | **Analysis method** | **Number of syndromes** | **Syndrome labels** |
| --- | --- | --- | --- | --- | --- | --- | --- | --- | --- | --- | --- | --- | --- | --- |
| Aalten 2007 | 2188 | 12 | Europe | Cross-sectional | 76.7 (7.8)  Range: 40 - 97 | 1609 (68.4%) |  | 17.8 (5.9)  Range: 0-29 | Outpatient | Not stated | 12 | PCA | 4 | • Hyperactivity (AGI, DIS, IRR, AMB) • Psychosis (DEL, HAL, NBD) • Affective (DEP, ANX) • Apathy (APA, APP) |
| Archer 2007 | 208 | 1 | UK | Cross-sectional | 81.2 (6.38)  Range: 67-96 | 152 (73%) | 10.36 (2.22) range: 5-21 | 12.64 | All patients recruited through old age psychiatry services and nursing homes  128 (62%) community dwelling 80 (38%) in long term care | NINCDS-ADRDA  (with modification to allow inclusion of those with age of onset >90 years) | 10 | PCA | 3 | • Agitation/apathy (IRR, AGI, APA, DIS, AMB) • Psychosis (HAL, DEL) • Affect (EUP, ANX, DEP) |
| Chen 2012 | 96 | 1 | Taiwan | Cross-sectional | 77.7 (7.8) range: 54-92 | 75 (78.1%) | 4.8 (5.1) range: 0-19 | 17.1 (5.2) range: 6-27 | Geriatric outpatient at teaching hospital | DSM-IV-TR and NINCDS-ADRDA | 12 | PCA | 5 | • Component 1 (DEL, AGI, ANX, IRR) • Component 2 (EUP, DIS, AMB • Component 3 (DEP, APA) • Component 4 (HAL, NBD) • Component 5 (APP) |
| Connors 2018 | 447 | 9 memory clinics | Australia | Longitudinal (3 years) observational study  Yearly visits | 78.6 (7.4) | 225 (50.3%) | Post secondary education: 151 (33.8%) | 21.1 (5.3) | Memory clinic | DSM-IV | 10 | PCA; Multi-group CFA | 3 or 4 | Three components • Component 1 (AGI, DEP, ANX, APA, IRR) • Component 2 (DEL, HAL) • Component 3 (EUP, DIS, AMB)  Four compjnents • Component 1 (AGI, DEP, ANX, IRR) • Component 2 (DEL, HAL) • Component 3 (APA, DIS, AMB) • Component 4 (EUP)  Multi-group CFA • Factor 1 (DEL, HAL) • Factor 2 (DEP, ANX, AGI, IRR) • Factor 3 (DIS, AMB, APA) |
| Cummings 2006 | 120 | Multicenter | USA | Cross-sectional | 76.9 (7.4) | 68 (57%) |  | 17.6 (4.6) | Outpatient | NINCDS-ADRDA | 12 | PCA | 5 | • Component 1 (DEL, HAL, NBD) • Component 2 (AGI, IRR, AMB) • Component 3 (DEP, ANX, APA) • Component 4 (EUP, DIS) • Component 5 (APP) |
| Dennehy 2013 | 581 | State registry | USA | Cross-sectional | 84 (8.2) | 420 (72.3%) | Less than high school: 232 (43.2%) High school or more: 305 (56.8%) |  | Statewide registry of residents with AD or related disorders | ICD-9-CM | 12 | CFA | 4 | • Hyperactivity (AGI, DIS, IRR, AMB) • Psychosis (DEL, HAL, NBD) • Affective (DEP, ANX) • Apathy (APA, APP) |
| Frisoni 1999 | 162 | 1 | Italy | Cross-sectional | 76.4 (8.5) range: 55-96 | 120 (74%) | 5.7 (3.1) range: 2-19 | 13.3 (6.9) range: 0-24 | Alzheimer's unit | NINCDS-ADRDA | 10 | PCA | 3 | • Mood (ANX, DEP, APA) • Psychosis (AGI, HAL, DEL, IRR) • Frontal (DIS, EUP)  AMB loaded equally well onto Psychosis and Frontal |
| Garre-Olmo 2010 | 491 | 1 | Spain | Longitudinal (2 years)  Bi-annual visits (every 6 mths) | 75.2 (6.6) | 348 (70.9%) | Illiterate: 55 (11.2%) Up to 8 years: 315 (64.2%) 9 years or more: 78 (15.9%) |  | Memory clinic | NINCDS-ADRDA | 10 | EFA; CFA | 3 | Baseline • Component 1 (DEL, HAL) • Component 2 (IRR, AGI, DEP, ANX, AMB, APA) • Component 3 (DIS, EUP)  6 month • Component 1 (DEL, HAL, ANX, DEP) • Component 2 (IRR, AGI, APA) • Component 3 (AMB, DIS, EUP)  12 month • Component 1 (DEL, HAL, APA, AMB) • Component 2 (IRR, AGI, DEP, ANX) • Component 3 (DIS, EUP)  18 month • Component 1 (DEL, HAL, ANX, AMB) • Component 2 (IRR, AGI, DEP, APA) • Component 3 (DIS, EUP)  24 month • Component 1 (DEL, HAL, APA, AMB) • Component 2 (IRR, AGI, DEP, ANX) • Component 3 (DIS, EUP)  From CFA best fitting model • Psychotic (DEL, HAL) • Emotional (AGI, IRR, DEP, ANX) • Behaviour (EUP, DIS, AMB, APA) |
| Gauthier 2005 | 252 | 32 | USA | Cross-sectional | Memantine: 75.9 (8.40)  Placebo: 76.3 (7.76) | 170 (67.5%) |  | Memantine: 7.7 (3.72)  Placebo: 8.1 (3.57) | Unclear | DSM-IV or NINCDS-ADRDA | 12 | PCA | 3 | • Hyperactivity (AGI, DEP, ANX, IRR, APA, APP) • Psychosis (DEL, HAL) • Mood/apathy (EUP, DIS, APA) |
| Germain 2009 | 1091 | 29 specialist outpatient clinics across 12 European countries | Europe | Cross-sectional | 78.6 (7.6) | 701 (64.2%) | 7.9 (4.7) | 20.4 (3.9) | Specialist outpatient clinics. All provided specialist secondary or tertiary care for patients with cognitive disorders | NINCDS-ADRDA | 12 | PCA | 5 | Based on cutoff • Component 1 (DEL, AGI, DEP, ANX, APA, DIS, IRR, AMB, NBD) • Component 2 (DEP, EUP, DIS) • Component 3 (DEL, HAL) • Component 4 (AGI, IRR, APP) • Component 5 (EUP, AMB, NBD) |
| Hollingworth 2006 | 1120 | 4 | UK (Wales, England) and Republic of Ireland | Cross-sectional | 81.2 (6.5)  Range: 62-99 | 786 (70.2%) | 10.4 (2.7)  Range: 0-23 | 12.8 (9)  Range: 0-28 | Volunteers identified through a variety of sources, including contact with clinical services, AD support groups, media advertising, and residential/nursing home residents. | NINCDS-ADRDA | 12 | PCA | 4 | • Behavioural dyscontrol (EUP, DIS, AMB, NBD, APP) • Psychosis (DEL, HAL) • Mood (DEP, ANX, APA) • Agitation (AGI, IRR) |
| Hwang 2017 | 149 | 1 | South Korea | Cross-sectional | With hypertension: 70.9 (7.4)  Without hypertension: 73.5 (7.5) | 94 (63.1%) | With hypertension: 9.4 (7.4) Without hypertension: 10.5 (5) | With hypertension: 23.3 (5.3)  Without hypertension: 22.6 (4.7) | Veterans health service | NINCDS-ADRDA | 12 | EFA | 3 | • Factor 1 (AGI, DIS, HAL, DEL, IRR) • Factor 2 (EUP, NBD, AMB) • Factor 3 (APP, APA, DEP, ANX) |
| Kang 2010a | 299 | 56 hospitals | South Korea | Cross-sectional | Full CREDOS cohort: 73.2 (7.7) | Full credos cohort: 541 (69.5%) | Full CREDOS cohort: 6.8 (5.4) | Total study sample (n = 600): 18.5 (5.3) | Hospital | NINCDS-ADRDA | 12 | EFA | 4 | • Hyperactivity (AGI, DIS, IRR) • Affect (DEP, ANX) • Psychosis (DEL, HAL) • Apathy/vegetative symptoms (APA, NBD, APP) |
| Kang 2010b | 301 | 56 hospitals | South Korea | Cross-sectional | Full CREDOS cohort: 73.2 (7.7) | Full credos cohort: 541 (69.5%) | Full CREDOS cohort: 6.8 (5.4) | Total study sample (n = 600): 18.5 (5.3) | Hospital | NINCDS-ADRDA | 12 | CFA | 4 | Best fitting model • Hyperactivity (AGI, DIS, IRR) • Affect (DEP, ANX) • Psychosis (DEL, HAL) • Apathy/vegetative symptoms (APA, NBD, APP) |
| Kazui 2016 | 1091 | 7 specialised centers for dementia | Japan | Cross-sectional | 76.9 (8.7) | 752 (68.9%) | 10.8 (2.8) | 18.9 (5.4) | Specialist dementia centers | NINCDS-ADRDA | 12 | PCA | 3 | • Component 1 (DEL, AGI, DEP, ANX, DIS, IRR) • Component 2 (HAL, APA, AMB, NBD, APP) • Component 3 (HAL, EUP, DIS) |
| Kim 2021 | 170 | 1 | South Korea | Cross-sectional | 76.5 (8.0) | 113 (66.5) | Never educated: 62 (36.5) Primary-junior high school: 60 (35.3) High school or above: 48 (28.2) | >=24: 98 (57.6)  18-23: 54 (31.8)  <=17: 18 (10.6) | Outpatient neurology clinic | DSM-IV or NINCDS ADRDA | 12 | PCA | 3 | • Hyperactivity (DIS, IRR, AGI)  • Psychosis symptoms (HAL, ANX, EUP, DEL, DEP)  • Physical behaviour symptoms (APP, APA, AMB, NBD) |
| Matsui 2006 | 140 | 3 | Japan | Cross-sectional | 72.4 (7.3) | 84 (60%) | 9.63 (0.82) | 20.3 (4.2) | Hospital | NINCDS-ADRDA | 10 | PCA | 3 | • Psychosis (DEL, HAL, ANX, AGI, DIS, IRR, AMB) • Mood (APA, DEP) • Euphoria (EUP) |
| Mirakhur 2004 | 435 | Unclear | UK (Northern Ireland) | Cross-sectional | 78 (7.5) | 66% | No education beyond primary: 9% Educated to secondary level: 84% Educated to tertiary level: 9% | 13 (9.2) | Old age psychiatry and elderly memory care outpatient clinics | NINCDS-ADRDA | 12 | PCA | 4 | • Affect (DEP, ANX, IRR, AGI) • Physical behaviour (APA, AMB, NBD, APP) • Psychosis (DEL, HAL) • Hypomania (DIS, EUP) |
| Nagata 2016 | 414 | 45 (26 university clinics, 7 Veterans Affairs medical centers, 12 private-practice) | USA | Cross-sectional | 77.9 (7.5)  Range: 51-103 | 235 (53%) | GED/High school diploma: 142 (33.7%) Did not complete high school: 101 (24%) < 4 years college: 90 (21.4%) >= 4 years of college: 73 (17.3%) Other/unknown: 14 (3.6%) | 15 (5.8)  Range: 4- 29 | University hospitals, VA medical centers, private practice | DSM-IV | 12 | PCA | 4 | • Aggressiveness (AGI, IRR) • Apathy and eating problem (APA, APP) • Psychosis (DEL, HAL) • Emotion and disinhibition (DEP, EUP, DIS) |
| Poletti 2013 | 140 | 1 | Italy | Cross-sectional | 80.3 (6.11) | 101 (72.1%) | 5.33 (3.16) | 15.56 (5.39) | Dementia outpatient clinic | NINCDS-ADRDA | 12 | PCA | 4 | • Disinhibition (DEL, AGI, DIS, IRR) • Affective (DEP, APA, ANX) • Sensory-motor (HAL, NBD, APP, AMB) • Euphoric (EUP) |
| Proitsi 2011 | 1850 | Multinational | UK (Wales, Northern Ireland, England), Republic of Ireland, Greece | Cross-sectional | 79.1 (6.7)  Range: 63-99 | 68.90% |  | 12.7 (8.5)  Range: 0 - 29 | All participants recruited through secondary care services | NINCDS-ADRDA | 10 | CFA | 4 | • Behavioural dyscontrol (DIS, EUP, APA, AMB) • Psychosis (DEL, HAL) • Mood (DEP, ANX) • Agitation (AGI, IRR, DIS) |
| Scassellati 2020 | 362 | 1 | Italy | Cross-sectional | 80.5 (7) | 241 (66.6%) | 6 (3.6) | 11.7 (7.3) | Alzheimer's unit | NINCDS-ADRDA | 12 | PCA | 4 | • Mood (DEP, ANX, APA) • Hyperactivity (AGI, IRR, APP) • Psychosis (DEL, HAL, AMB, NBD) • Frontal (EUP, DIS) |
| Spalletta 2010 | 1015 | 5 | Italy | Cross-sectional | 74.6 (0.2)  Range: 45 - 96 | 723 (71.2%) | 6.2 (0.1)  range: 0 - 19 | 18.3 (0.2) | Outpatient memory clinic | NINCDS-ADRDA | 10 | PCA | 5 | • Component 1 (AGI, IRR, AMB) • Component 2 (DEL, HAL) • Component 3 (DEP, ANX) • Component 4 (EUP, DIS) • Component 5 (APA) |
| Starr 2007 | 556 | 1 | UK (Scotland) | Cross-sectional | 77.3 | 389 (70%) |  | 19.2 | Tertiary-referral memory treatment centre | NINCDS-ADRDA | 10 | PCA | 3 | Not reported |
| Vilalta-Franch 2010 | 491 | Unclear | Spain | Longitudinal (1 year) | 75.2 (6.6)  range: 52-89 | 70.90% | 5.9 (3.7)  range: 0 -21 | 17.1 (4.1)  range: 6 - 27 | Memory clinic | NINCDS-ADRDA | 12 but only 10 domains reported. So treat as NPI-10 study. | PCA | 3 | Baseline • Component 1 (AGI, DEP, ANX, APA, IRR) • Component 2 (DEL, HAL, AMB) • Component 3 (EUP, DIS)  One year follow up • Component 1 (DEP, ANX, EUP, IRR) • Component 2 (DEL, HAL, APA) • Component 3 (EUP, DIS, AMB) |
| Wang 2012 | 219 | 1 | China | Cross-sectional | 72 (9) | 153 (69.9%) |  | 18.6 (8.1) | Tertiary hospital |  | 12 | EFA | 5 | • Factor 1 (AMB, HAL, DEL, AGI) • Factor 2 (DEP, NBD) • Factor 3 (IRR, DIS, EUP) • Factor 4 (ANX, APA) • Factor 5 (APP) |

PCA, principal component analysis; EFA, exploratory factor analysis; CFA, confirmatory factor analysis; DEL, delusions; HAL, hallucinations, AGI, agitation; DEP, depression; ANX, anxiety; EUP, euphoria; APA, apathy; DIS, disinhibition; IRR, irritability; AMB, aberrant motor behaviour; NBD, night-time behavioural disturbances; APP, appetite and eating abnormalities.
