# Supplementary material for: Neuropsychiatric Inventory domains cluster into neuropsychiatric syndromes in Alzheimer's disease: A systematic review and meta‐analysis
Source: Brain Behav. 2022 Aug 8;12(9):e2734. doi: 10.1002/brb3.2734 (PMC9480932; doi:10.1002/brb3.2734)
Supplement: Supplementary file 2 — Supplemental Material 2: COSMIN risk of bias ratings [file BRB3-12-e2734-s001.docx]

Supplemental material 2: COSMIN risk of bias ratings

| **Study** | **SV1** | **SV2** | **SV3** | **SV4** | **IC1** | **IC2** | **IC3** | **IC4** | **CCV1** | **CCV2** | **CCV3** |
| --- | --- | --- | --- | --- | --- | --- | --- | --- | --- | --- | --- |
| Aalten 2007 | Adequate. PCA was used. | NA | Very good. Sample size was n = 2188 | No other important methodological flaws | Very good. Cronbach's alpha was reported for each syndrome | Adequate. Cronbach's alpha was only reported for two syndromes. | NA | NA | NA | NA | NA |
| Archer 2007 | Adequate. PCA was used. | NA | Very good. Sample size was n = 208 | No other important methodological flaws | Inadequate. Measures of internal consistency were not reported. | Inadequate. Measures of internal consistency were not reported. | NA | NA | NA | NA | NA |
| Chen 2012 | Adequate. PCA was used. | NA | Adequate. Sample size was n = 96 | Doubtful. Orthogonal rotation may not be appropriate as no strong reason to assume syndromes are uncorrelated. | Inadequate. Measures of internal consistency were not reported. | Inadequate. Measures of internal consistency were not reported. | NA | NA | NA | NA | NA |
| Connors 2018 | Very good. Multi-group CFA was used | NA | Very good. Sample size was n = 447 | No other important methodological flaws | Inadequate. Measures of internal consistency were not reported. | Inadequate. Measures of internal consistency were not reported. | NA | NA | Very good. Same sample was followed up at all visits. | Very good. Authors tested for longitudinal invariance using appropriate methodology. | Very good. Sample size was sufficiently large. |
| Cummings 2006 | Adequate. PCA was used. | NA | Very good. Sample size was n = 120 | Inadequate. Loadings, eigenvalues, and variance explained not reported | Inadequate. Measures of internal consistency were not reported. | Inadequate. Measures of internal consistency were not reported. | NA | NA | NA | NA | NA |
| Dennehy 2013 | Very good. Confirmatory factor analysis was performed. | NA | Very good. Sample size was n = 581 | No other important methodological flaws | Inadequate. Cronbach's alpha calculated entire NPI but not each syndrome subscale. | Very good. Cronbach's alpha reported. | NA | NA | NA | NA | NA |
| Frisoni 1999 | Adequate. PCA was used. | NA | Very good. Sample size was n = 162 | Doubtful. Orthogonal rotation may not be appropriate as no strong reason to assume syndromes are uncorrelated. | Inadequate. Measures of internal consistency were not reported. | Inadequate. Measures of internal consistency were not reported. | NA | NA | NA | NA | NA |
| Garre-Olmo 2010 | Very good. Confirmatory factor analysis was performed. | NA | Very good. Sample size was n = 491 | No other important methodological flaws | Inadequate. Measures of internal consistency were not reported. | Inadequate. Measures of internal consistency were not reported. | NA | NA | Doubtful. Unclear if participants who remained at follow up visits systematically differed from those at baseline. | Inadequate. Tests of longitudinal invariance should be used. | Very good. Sample size was sufficiently large. |
| Gauthier 2005 | Adequate. PCA was used. | NA | Very good. Sample size was n = 252 | Doubtful. Orthogonal rotation may not be appropriate as no strong reason to assume syndromes are uncorrelated. | Inadequate. Measures of internal consistency were not reported. | Inadequate. Measures of internal consistency were not reported. | NA | NA | NA | NA | NA |
| Germain 2009 | Adequate. PCA was used. | NA | Very good. Sample size was n = 1091 | Doubtful. Orthogonal rotation may not be appropriate as no strong reason to assume syndromes are uncorrelated. | Inadequate. Measures of internal consistency were not reported. | Inadequate. Measures of internal consistency were not reported. | NA | NA | NA | NA | NA |
| Hollingworth 2006 | Adequate. PCA was used. | NA | Very good. Sample size was n = 1120 | No other important methodological flaws | Inadequate. Measures of internal consistency were not reported. | Inadequate. Measures of internal consistency were not reported. | NA | NA | NA | NA | NA |
| Hwang 2017 | Adequate. EFA was used. | NA | Very good. Sample size was n = 149 | Doubtful. Orthogonal rotation may not be appropriate as no strong reason to assume syndromes are uncorrelated. | Very good. Cronbach's alpha was computed for each syndrome subscale. | Very good. Cronbach's alpha was computed. | NA | NA | NA | NA | NA |
| Kang 2010a | Adequate. EFA was used. | NA | Very good. Sample size was n = 299 | No other important methodological flaws | Inadequate. Measures of internal consistency were not reported. | Inadequate. Measures of internal consistency were not reported. | NA | NA | NA | NA | NA |
| Kang 2010b | Very good. Confirmatory factor analysis was performed. | NA | Very good. Sample size was n = 301 | No other important methodological flaws | Inadequate. Measures of internal consistency were not reported. | Inadequate. Measures of internal consistency were not reported. | NA | NA | NA | NA | NA |
| Kazui 2016 | Adequate. PCA was used. | NA | Very good. Sample size was n = 1091 | Doubtful. Orthogonal rotation may not be appropriate as no strong reason to assume syndromes are uncorrelated. | Inadequate. Measures of internal consistency were not reported. | Inadequate. Measures of internal consistency were not reported. | NA | NA | NA | NA | NA |
| Kim 2021 | Adequate. PCA was used. | NA | Very good. Sample size was n = 170 | Doubtful. Orthogonal rotation may not be appropriate as no strong reason to assume syndromes are uncorrelated. | Very good. Internal consistency statistic calculated for each unidimensional scale or subscale | Very good. Cronbach's alpha reported. | NA | NA | NA | NA | NA |
| Matsui 2006 | Adequate. PCA was used. | NA | Very good. Sample size was n = 140 | Doubtful. Orthogonal rotation may not be appropriate as no strong reason to assume syndromes are uncorrelated. No information on criteria used to select number of components, or what is a sufficient loading. | Inadequate. Measures of internal consistency were not reported. | Inadequate. Measures of internal consistency were not reported. | NA | NA | NA | NA | NA |
| Mirakhur 2004 | Adequate. PCA was used. | NA | Very good. Sample size was n = 435 | No other important methodological flaws | Inadequate. Measures of internal consistency were not reported. | Inadequate. Measures of internal consistency were not reported. | NA | NA | NA | NA | NA |
| Nagata 2016 | Adequate. PCA was used. | NA | Very good. Sample size was n = 414 | No other important methodological flaws | Inadequate. Measures of internal consistency were not reported. | Inadequate. Measures of internal consistency were not reported. | NA | NA | NA | NA | NA |
| Poletti 2013 | Adequate. PCA was used. | NA | Very good. Sample size was n = 140 | Doubtful. Orthogonal rotation may not be appropriate as no strong reason to assume syndromes are uncorrelated. | Inadequate. Measures of internal consistency were not reported. | Inadequate. Measures of internal consistency were not reported. | NA | NA | NA | NA | NA |
| Proitsi 2011 | Very good. Confirmatory factor analysis was performed. | NA | Very good. Sample size was n = 1850 | No other important methodological flaws | Inadequate. Measures of internal consistency were not reported. | Inadequate. Measures of internal consistency were not reported. | NA | NA | Adequate. Although unclear whether samples were similar for relevant characteristics except group variable, model included adjustments for other important covariates. | Very good. The authors used MIMIC model to test for direct effects of covariates on latent variables and manifest variables. | Very good. Sample size was sufficiently large. |
| Scassellati 2020 | Adequate. PCA was used. | NA | Very good. Sample size was n = 362 | Doubtful. Orthogonal rotation may not be appropriate as no strong reason to assume syndromes are uncorrelated. | Inadequate. Measures of internal consistency were not reported. | Inadequate. Measures of internal consistency were not reported. | NA | NA | NA | NA | NA |
| Spalletta 2010 | Adequate. PCA was used. | NA | Very good. Sample size was n = 1015 | Doubtful. Orthogonal rotation may not be appropriate as no strong reason to assume syndromes are uncorrelated. Loading cut-off used is larger than the convention. | Inadequate. Measures of internal consistency were not reported. | Inadequate. Measures of internal consistency were not reported. | NA | NA | NA | NA | NA |
| Starr 2007 | Adequate. PCA was used. | NA | Very good. Sample size was n = 556 | Doubtful. Orthogonal rotation may not be appropriate as no strong reason to assume syndromes are uncorrelated. Loading cut-off used is larger than the convention. | Inadequate. Measures of internal consistency were not reported. | Inadequate. Measures of internal consistency were not reported. | NA | NA | NA | NA | NA |
| Vilalta-Franch 2010 | Adequate. PCA was used. | NA | Very good. Sample size was n = 491 | No other important methodological flaws | Inadequate. Measures of internal consistency were not reported. | Inadequate. Measures of internal consistency were not reported. | NA | NA | Doubtful. Unclear if participants who remained at follow up visits systematically differed from those at baseline. | Inadequate. Tests of longitudinal invariance should be used. | Very good. Sample size was sufficiently large. |
| Wang 2012 | Adequate. EFA was used. | NA | Very good. Sample size was n = 219 | Doubtful. Orthogonal rotation may not be appropriate as no strong reason to assume syndromes are uncorrelated. | Inadequate. Cronbach's alpha calculated for entire NPI but not each syndrome subscale. | Very good. Cronbach's alpha reported. | NA | NA | NA | NA | NA |

SV, structural validity; IC, internal consistency; CCV, cross-cultural validity/measurement invariance.
