# Supplementary material for: Neuropsychiatric Inventory domains cluster into neuropsychiatric syndromes in Alzheimer's disease: A systematic review and meta‐analysis
Source: Brain Behav. 2022 Aug 8;12(9):e2734. doi: 10.1002/brb3.2734 (PMC9480932; doi:10.1002/brb3.2734)
Supplement: Supplementary file 3 — Supplemental Material 3: The data presented below are copied verbatim from the email correspondences with study corresponding authors to preserve authenticity unless otherwise specified. Notes made by the study author (SKWH) are highlighted in yellow. [file BRB3-12-e2734-s004.docx]

**Supplemental material 3**

The data presented below are copied verbatim from the email correspondences with study corresponding authors to preserve authenticity, unless otherwise specified. Notes made by the study author (SKWH) are highlighted in yellow.

**1. OBSERVED CORRELATION MATRICES**

**Connors et al (2018)**

Sample size: 447

|  |  | **1 Delusions** | **2 Hallucinations** | **3 Agitation** | **4 Depression** | **5 Anxiety** | **6 Euphoria** | **7 Apathy** | **8 Disinhibition** | **9 Irritability** | **10 Aberrant Motor** |
| --- | --- | --- | --- | --- | --- | --- | --- | --- | --- | --- | --- |
| **1 Delusions** | Pearson Correlation | 1.000 | .518** | .440** | .179** | .344** | 0.028 | .156** | .199** | .385** | .151** |
|  | Sig. (2-tailed) |  | 0.000 | 0.000 | 0.000 | 0.000 | 0.559 | 0.001 | 0.000 | 0.000 | 0.001 |
| **2 Hallucinations** | Pearson Correlation | .518** | 1.000 | .264** | .296** | .289** | 0.005 | 0.064 | .122** | .296** | .252** |
|  | Sig. (2-tailed) | 0.000 |  | 0.000 | 0.000 | 0.000 | 0.917 | 0.177 | 0.010 | 0.000 | 0.000 |
| **3 Agitation** | Pearson Correlation | .440** | .264** | 1.000 | .262** | .599** | .178** | .250** | .296** | .663** | .402** |
|  | Sig. (2-tailed) | 0.000 | 0.000 |  | 0.000 | 0.000 | 0.000 | 0.000 | 0.000 | 0.000 | 0.000 |
| **4 Depression** | Pearson Correlation | .179** | .296** | .262** | 1.000 | .404** | .271** | .261** | .218** | .364** | .264** |
|  | Sig. (2-tailed) | 0.000 | 0.000 | 0.000 |  | 0.000 | 0.000 | 0.000 | 0.000 | 0.000 | 0.000 |
| **5 Anxiety** | Pearson Correlation | .344** | .289** | .599** | .404** | 1.000 | .129** | .270** | .219** | .551** | .412** |
|  | Sig. (2-tailed) | 0.000 | 0.000 | 0.000 | 0.000 |  | 0.006 | 0.000 | 0.000 | 0.000 | 0.000 |
| **6 Euphoria** | Pearson Correlation | 0.028 | 0.005 | .178** | .271** | .129** | 1.000 | 0.021 | .342** | .189** | .193** |
|  | Sig. (2-tailed) | 0.559 | 0.917 | 0.000 | 0.000 | 0.006 |  | 0.663 | 0.000 | 0.000 | 0.000 |
| **7 Apathy** | Pearson Correlation | .156** | 0.064 | .250** | .261** | .270** | 0.021 | 1.000 | .152** | .260** | .266** |
|  | Sig. (2-tailed) | 0.001 | 0.177 | 0.000 | 0.000 | 0.000 | 0.663 |  | 0.001 | 0.000 | 0.000 |
| **8 Disinhibition** | Pearson Correlation | .199** | .122** | .296** | .218** | .219** | .342** | .152** | 1.000 | .407** | .370** |
|  | Sig. (2-tailed) | 0.000 | 0.010 | 0.000 | 0.000 | 0.000 | 0.000 | 0.001 |  | 0.000 | 0.000 |
| **9 Irritability** | Pearson Correlation | .385** | .296** | .663** | .364** | .551** | .189** | .260** | .407** | 1.000 | .423** |
|  | Sig. (2-tailed) | 0.000 | 0.000 | 0.000 | 0.000 | 0.000 | 0.000 | 0.000 | 0.000 |  | 0.000 |
| **10 Aberrant Motor** | Pearson Correlation | .151** | .252** | .402** | .264** | .412** | .193** | .266** | .370** | .423** | 1.000 |
|  | Sig. (2-tailed) | 0.001 | 0.000 | 0.000 | 0.000 | 0.000 | 0.000 | 0.000 | 0.000 | 0.000 |  |

** Correlation is significant at the 0.01 level (2-tailed).

**Garre-Olmo et al (2010)**

Sample size: 491

| **Correlations** | | | | | | | | | | | |
| --- | --- | --- | --- | --- | --- | --- | --- | --- | --- | --- | --- |
|  | | TNPIA | TNPIB | TNPIC | TNPID | TNPIE | TNPIF | TNPIG | TNPIH | TNPII | TNPIJ |
| TNPIA | Pearson Correlation | 1 | ,468^**^ | ,357^**^ | ,205^**^ | ,125^**^ | ,088 | ,157^**^ | ,219^**^ | ,300^**^ | ,210^**^ |
|  | Sig. (2-tailed) |  | ,000 | ,000 | ,000 | ,006 | ,051 | ,000 | ,000 | ,000 | ,000 |
|  | N | 491 | 491 | 491 | 491 | 491 | 491 | 491 | 491 | 491 | 491 |
| TNPIB | Pearson Correlation | ,468^**^ | 1 | ,241^**^ | ,188^**^ | ,015 | ,030 | ,203^**^ | ,164^**^ | ,192^**^ | ,246^**^ |
|  | Sig. (2-tailed) | ,000 |  | ,000 | ,000 | ,740 | ,505 | ,000 | ,000 | ,000 | ,000 |
|  | N | 491 | 491 | 491 | 491 | 491 | 491 | 491 | 491 | 491 | 491 |
| TNPIC | Pearson Correlation | ,357^**^ | ,241^**^ | 1 | ,316^**^ | ,176^**^ | ,105^*^ | ,347^**^ | ,289^**^ | ,513^**^ | ,318^**^ |
|  | Sig. (2-tailed) | ,000 | ,000 |  | ,000 | ,000 | ,020 | ,000 | ,000 | ,000 | ,000 |
|  | N | 491 | 491 | 491 | 491 | 491 | 491 | 491 | 491 | 491 | 491 |
| TNPID | Pearson Correlation | ,205^**^ | ,188^**^ | ,316^**^ | 1 | ,369^**^ | ,100^*^ | ,333^**^ | ,163^**^ | ,348^**^ | ,134^**^ |
|  | Sig. (2-tailed) | ,000 | ,000 | ,000 |  | ,000 | ,027 | ,000 | ,000 | ,000 | ,003 |
|  | N | 491 | 491 | 491 | 491 | 491 | 491 | 491 | 491 | 491 | 491 |
| TNPIE | Pearson Correlation | ,125^**^ | ,015 | ,176^**^ | ,369^**^ | 1 | ,055 | ,062 | ,027 | ,203^**^ | ,162^**^ |
|  | Sig. (2-tailed) | ,006 | ,740 | ,000 | ,000 |  | ,221 | ,173 | ,554 | ,000 | ,000 |
|  | N | 491 | 491 | 491 | 491 | 491 | 491 | 491 | 491 | 491 | 491 |
| TNPIF | Pearson Correlation | ,088 | ,030 | ,105^*^ | ,100^*^ | ,055 | 1 | ,024 | ,286^**^ | ,139^**^ | ,159^**^ |
|  | Sig. (2-tailed) | ,051 | ,505 | ,020 | ,027 | ,221 |  | ,600 | ,000 | ,002 | ,000 |
|  | N | 491 | 491 | 491 | 491 | 491 | 491 | 491 | 491 | 491 | 491 |
| TNPIG | Pearson Correlation | ,157^**^ | ,203^**^ | ,347^**^ | ,333^**^ | ,062 | ,024 | 1 | ,095^*^ | ,323^**^ | ,261^**^ |
|  | Sig. (2-tailed) | ,000 | ,000 | ,000 | ,000 | ,173 | ,600 |  | ,035 | ,000 | ,000 |
|  | N | 491 | 491 | 491 | 491 | 491 | 491 | 491 | 491 | 491 | 491 |
| TNPIH | Pearson Correlation | ,219^**^ | ,164^**^ | ,289^**^ | ,163^**^ | ,027 | ,286^**^ | ,095^*^ | 1 | ,273^**^ | ,249^**^ |
|  | Sig. (2-tailed) | ,000 | ,000 | ,000 | ,000 | ,554 | ,000 | ,035 |  | ,000 | ,000 |
|  | N | 491 | 491 | 491 | 491 | 491 | 491 | 491 | 491 | 491 | 491 |
| TNPII | Pearson Correlation | ,300^**^ | ,192^**^ | ,513^**^ | ,348^**^ | ,203^**^ | ,139^**^ | ,323^**^ | ,273^**^ | 1 | ,311^**^ |
|  | Sig. (2-tailed) | ,000 | ,000 | ,000 | ,000 | ,000 | ,002 | ,000 | ,000 |  | ,000 |
|  | N | 491 | 491 | 491 | 491 | 491 | 491 | 491 | 491 | 491 | 491 |
| TNPIJ | Pearson Correlation | ,210^**^ | ,246^**^ | ,318^**^ | ,134^**^ | ,162^**^ | ,159^**^ | ,261^**^ | ,249^**^ | ,311^**^ | 1 |
|  | Sig. (2-tailed) | ,000 | ,000 | ,000 | ,003 | ,000 | ,000 | ,000 | ,000 | ,000 |  |
|  | N | 491 | 491 | 491 | 491 | 491 | 491 | 491 | 491 | 491 | 491 |
| **. Correlation is significant at the 0.01 level (2-tailed). | | | | | | | | | | | |
| *. Correlation is significant at the 0.05 level (2-tailed). | | | | | | | | | | | |

Items are indexed as: TNPIA. Delusions; TNPIB. Hallucinations; TNPIC. Agitation; TNPID. Depression; TNPIE. Anxiety; TNPIF. Euphoria; TNPIG. Apathy; TNPIH. Disinhibiton; TNPII. Irritability; TNPIJ. Aberrant motor behaviour.

**Germain et al (2009)**

Sample size: 1076

The dataset was shared with the study author (SHKW) who computed the correlation matrix for n = 1076 participants with complete data for all NPI-12 items.

|  | DEL | HAL | AGI | DEP | ANX | EUP | APA | DIS | IRR | AMB | NBD | APP |
| --- | --- | --- | --- | --- | --- | --- | --- | --- | --- | --- | --- | --- |
| DEL | 1 | 0.346 | 0.253 | 0.229 | 0.106 | 0.041 | 0.112 | 0.165 | 0.157 | 0.146 | 0.155 | 0.13 |
| HAL | 0.346 | 1 | 0.14 | 0.085 | 0.022 | 0.093 | 0.068 | 0.123 | 0.106 | 0.128 | 0.191 | 0.083 |
| AGI | 0.253 | 0.14 | 1 | 0.182 | 0.3 | 0.103 | 0.204 | 0.258 | 0.478 | 0.225 | 0.184 | 0.122 |
| DEP | 0.229 | 0.085 | 0.182 | 1 | 0.368 | 0.095 | 0.266 | 0.127 | 0.189 | 0.128 | 0.171 | 0.188 |
| ANX | 0.106 | 0.022 | 0.3 | 0.368 | 1 | 0.08 | 0.215 | 0.136 | 0.208 | 0.12 | 0.24 | 0.133 |
| EUP | 0.041 | 0.093 | 0.103 | 0.095 | 0.08 | 1 | 0.076 | 0.285 | 0.183 | 0.114 | 0.073 | 0.054 |
| APA | 0.112 | 0.068 | 0.204 | 0.266 | 0.215 | 0.076 | 1 | 0.184 | 0.169 | 0.249 | 0.186 | 0.175 |
| DIS | 0.165 | 0.123 | 0.258 | 0.127 | 0.136 | 0.285 | 0.184 | 1 | 0.289 | 0.244 | 0.11 | 0.15 |
| IRR | 0.157 | 0.106 | 0.478 | 0.189 | 0.208 | 0.183 | 0.169 | 0.289 | 1 | 0.188 | 0.142 | 0.081 |
| AMB | 0.146 | 0.128 | 0.225 | 0.128 | 0.12 | 0.114 | 0.249 | 0.244 | 0.188 | 1 | 0.079 | 0.147 |
| NBD | 0.155 | 0.191 | 0.184 | 0.171 | 0.24 | 0.073 | 0.186 | 0.11 | 0.142 | 0.079 | 1 | 0.164 |
| APP | 0.13 | 0.083 | 0.122 | 0.188 | 0.133 | 0.054 | 0.175 | 0.15 | 0.081 | 0.147 | 0.164 | 1 |

DEL, delusions; HAL, hallucinations, AGI, agitation; DEP, depression; ANX, anxiety; EUP, euphoria; APA, apathy; DIS, disinhibition; IRR, irritability; AMB, aberrant motor behaviour; NBD, night-time behavioural disturbances; APP, appetite and eating abnormalities.

**Kang et al (2010)**

Sample size: 600

Items are indexed as: 1. Delusions; 2. Hallucinations; 3. Agitation; 4. Depression; 5. Anxiety; 6. Euphoria; 7. Apathy; 8. Disinhibiton; 9. Irritability; 10. Aberrant motor behaviour; 11. Night-time behavioural disturbances; 12. Appetite and eating abnormalities.

**Nagata et al (2016)**

Sample size: 414

|  | NPIATotal | NPIBTotal | NPICTotal | NPIDTotal | NPIETotal | NPIFTotal | NPIGTotal | NPIHTotal | NPIITotal | NPIJTotal | NPIKTotal | NPILTotal |
| --- | --- | --- | --- | --- | --- | --- | --- | --- | --- | --- | --- | --- |
| NPIATotal Pearson coefficient　　　　　　　P-value　　　　 Covariance　　　　　　　N |  | .290** .000 4.278 413 | .161** .001 2.321 414 | .203** .000 2.638 414 | .210* .000 3.195 414 | .075 .128 .431 413 | .029 .551 .448 413 | .102* .038 1.349 413 | .124* .012 1.924 413 | .174** .000 3.057 413 | .142** .004 2.383 411 | .055 .262 .806 413 |
| NPIBTotal Pearson coefficient　　　　　　　P-value　　　　　　　　Covariance　　　　　　　N | .290** .000 4.278 413 |  | .031 .535 .404 413 | .018 .722 .210 413 | .076 .122 1.068 413 | .079 .113 .414 412 | .165** .001 2.314 412 | .102* .038 1.247 412 | .028 .571 .400 412 | .204** .000 3.304 412 | .159** .001 2.466 410 | .141** .004 1.876 412 |
| NPICTotal Pearson coefficient　　　　　　　P-value　　　　　　Covariance　　　　　　N | .161** .001 2.321 414 | .310 .535 .404 413 |  | .173** .000 2.013 414 | .274** .000 3.736 414 | .116* .018 .598 413 | .190** .000 2.588 413 | .302** .000 3.581 413 | .621** .000 8.635 413 | .188** .001 2.642 413 | .161** .001 2.427 411 | .089 .071 1.160 413 |
| NPIDTotal Pearson coefficient　　　　　　　P-value　　　　　　　　Covariance　　　　　　　　N | .203** .000 2.638 414 | .018 .722 .210 413 | .173** .000 2.013 414 |  | .345** .000 4.257 414 | -.059 .230 '-.276 413 | .254** .000 3.139 413 | .096 .052 1.024 413 | .262** .000 3.290 413 | .108* .028 1.540 413 | .100* .043 1.357 411 | .205** .000 2.420 413 |
| NPIETotal Pearson coefficient　　　　　　　P-value　　　　　　　　Covariance　　　　　　　　N | .210** .000 3.195 414 | .076 .122 1.068 413 | .274** .000 3.736 414 | .345** .000 4.257 414 |  | .014 .771 .078 413 | .217** .000 3.135 413 | .170** .001 2.135 413 | .301** .000 4.419 413 | .248** .000 4.129 413 | .119* .016 1.894 411 | .108* .028 1.497 413 |
| NPIFTotal Pearson coefficient　　　　　　　P-value　　　　　　　　Covariance　　　　　　　　N | .075 .128 .431 413 | .078 .113 .414 412 | .116* .018 .598 413 | -.059 .230 '-.276 413 | .014 .771 .078 413 |  | .091 .064 .498 413 | .212** .000 1.007 412 | .049 .322 .272 412 | .120* .015 .757 412 | .078 .114 .472 410 | .083 .092 .435 412 |
| NPIGTotal Pearson coefficient　　　　　　　　P-value　　　　　　　　Covariance　　　　　　　　N | .029 .551  .449 413 | .165** .001 2.314 412 | .190** .000 2.588 413 | .254** .000 3.139 413 | .217** .000 3.135 413 | .091 .064 .498 413 |  | .181** .000 2.264 412 | .188** .000 2.766 412 | .196** .000 3.266 412 | .147** .003 2.350 410 | .198** .000 2.728 412 |
| NPIHTotal Pearson coefficient　　　　　　　P-value　　　　　　　　Covariance　　　　　　　　N | .102* .038 1.349 413 | .102* .038 1.247 412 | .302** .000 3.581 413 | .096 .052 1.024 413 | .170** .001 2.135 413 | .212** .000 1.007 412 | .181** .000 2.264 412 |  | .299** .000 3.813 413 | .320** .000 4.630 413 | .154** .002 2.123 411 | .140** .004 1.682 413 |
| NPIITotal Pearson coefficient　　　　　　　P-value　　　　　　　　Covariance　　　　　　　N | .124* .012 1.924 413 | .280 .571 .400 412 | .621** .000 8.635 413 | .262** .000 3.290 413 | .301** .000 4.419 413 | .049 .322  .272 412 | .188** .000 2.766 412 | .299** .000 3.813 413 |  | .214** .000 3.621 413 | .096 .051 1.561 411 | .067 .174 .942 413 |
| NPIJTotal Pearson coefficient　　　　　　P-value　　　　　　　Covariance　　　　　　　N | .174** .000 3.057 413 | .204** .000 3.304 412 | .168** .001 2.642 413 | .108* .028 1.540 413 | .248** .000 4.129 413 | .120* .015 .757 412 | .196** .000 3.266 412 | .320** .000 4.630 413 | .214** .000 3.621 413 |  | .239** .000 4.392 411 | .227** .000 3.616 413 |
| NPIKTotal Pearson coefficient　　　　　　　P-value　　　　　　　　Covariance　　　　　　　N | .142** .004 2.383 411 | .159** .001 2.466 410 | .161** .001 2.427 411 | .100* .043 1.357 411 | .119* .016 1.894 411 | .079 .114 .472 410 | .147** .003 2.350 410 | .154** .002 2.123 411 | .096 .051 1.561 411 | .239** .000 4.392 411 |  | .207** .000 3.124 411 |
| NPILTotal Pearson coefficient　　　　　　　P-value　　　　　　　　Covariance　　　　　　　　N | .055 .262 .806 413 | .141** .004 1.876 412 | .089 .071 1.160 413 | .205** .000 2.420 413 | .108* .028 1.497 413 | .083 .092 .435 412 | .198** .000 2.728 412 | .140** .004 1.682 413 | .067 .174 .942 413 | .227** .000 3.616 413 | .207** .000 3.124 411 |  |

*P<0.05, **P<0.01

NPI sub-items

NPIA: Delusion

NPIB: Hallucination

NPIC: Agitation

NPID: Depression

NPIE: Anxiety

NPIF: Euphoria/elation

NPIG: Apathy

NPIH: Disinhibition

NPII: Irritability

NPIJ: Aberrant motor behavior

NPIK: Sleep/nighttime behavior disorders

NPIL: Appetite/eating disorders

For the meta-analysis, we used n = 414 for the study sample size, as per the publication.

**Scassellati et al. (2020)**

Sample size: 307

| **Pearson Correlation r** | Delusions | Hallucination | Agitation | Depression | Anxiety | Euphoria | Apaty | Disinhibition | Irritability | Aberrant motor behaviour | Night-time behaviour disturbances | Appetite and eating disturbances |
| --- | --- | --- | --- | --- | --- | --- | --- | --- | --- | --- | --- | --- |
| Delusions | 1 | 0.394430916 | 0.410655673 | 0.317707925 | 0.258797247 | 0.013290628 | 0.064334655 | 0.205736558 | 0.317694495 | 0.228254153 | 0.11004163 | 0.221972662 |
| Hallucination | 0.394430916 | 1 | 0.259754154 | 0.104009112 | 0.055732479 | 0.188212208 | -0.018781943 | 0.086789927 | 0.202586295 | 0.153222407 | 0.241128033 | 0.065486131 |
| Agitation | 0.410655673 | 0.259754154 | 1 | 0.201377514 | 0.231450185 | 0.087448173 | 0.070853517 | 0.168089552 | 0.570800636 | 0.27101578 | 0.280149219 | 0.276454781 |
| Depression | 0.317707925 | 0.104009112 | 0.201377514 | 1 | 0.597517837 | 0.031590469 | 0.328968826 | 0.161904265 | 0.179569718 | 0.129673301 | 0.126951502 | 0.19801124 |
| Anxiety | 0.258797247 | 0.055732479 | 0.231450185 | 0.597517837 | 1 | 0.02464588 | 0.277818327 | 0.149283208 | 0.200731404 | 0.173715412 | 0.072981903 | 0.139686931 |
| Euphoria | 0.013290628 | 0.188212208 | 0.087448173 | 0.031590469 | 0.02464588 | 1 | -0.072487433 | 0.184864049 | 0.112300593 | 0.02208056 | 0.08631485 | 0.001983787 |
| Apathy | 0.064334655 | -0.018781943 | 0.070853517 | 0.328968826 | 0.277818327 | -0.072487433 | 1 | 0.052656533 | 0.09124161 | 0.113775224 | -0.002476819 | 0.268969973 |
| Disinhibition | 0.205736558 | 0.086789927 | 0.168089552 | 0.161904265 | 0.149283208 | 0.184864049 | 0.052656533 | 1 | 0.193332711 | 0.0706938 | 0.004070594 | 0.171103766 |
| Irritability | 0.317694495 | 0.202586295 | 0.570800636 | 0.179569718 | 0.200731404 | 0.112300593 | 0.09124161 | 0.193332711 | 1 | 0.269476321 | 0.187429732 | 0.183663798 |
| Aberrant motor behaviour | 0.228254153 | 0.153222407 | 0.27101578 | 0.129673301 | 0.173715412 | 0.02208056 | 0.113775224 | 0.0706938 | 0.269476321 | 1 | 0.157189794 | 0.101932729 |
| Night-time behaviour disturbances | 0.11004163 | 0.241128033 | 0.280149219 | 0.126951502 | 0.072981903 | 0.08631485 | -0.002476819 | 0.004070594 | 0.187429732 | 0.157189794 | 1 | 0.118509751 |
| Appetite and eating disturbances | 0.221972662 | 0.065486131 | 0.276454781 | 0.19801124 | 0.139686931 | 0.001983787 | 0.268969973 | 0.171103766 | 0.183663798 | 0.101932729 | 0.118509751 | 1 |

| **Sample size** | Delusions | Hallucination | Agitation | Depression | Anxiety | Euphoria | Apaty | Disinhibition | Irritability | Aberrant motor behaviour | Night-time behaviour disturbances | Appetite and eating disturbances |
| --- | --- | --- | --- | --- | --- | --- | --- | --- | --- | --- | --- | --- |
| Delusions | 307 | 307 | 307 | 307 | 306 | 307 | 307 | 307 | 307 | 307 | 307 | 307 |
| Hallucination | 307 | 307 | 307 | 307 | 306 | 307 | 307 | 307 | 307 | 307 | 307 | 307 |
| Agitation | 307 | 307 | 309 | 307 | 306 | 307 | 307 | 307 | 308 | 307 | 309 | 309 |
| Depression | 307 | 307 | 307 | 307 | 306 | 307 | 307 | 307 | 307 | 307 | 307 | 307 |
| Anxiety | 306 | 306 | 306 | 306 | 306 | 306 | 306 | 306 | 306 | 306 | 306 | 306 |
| Euphoria | 307 | 307 | 307 | 307 | 306 | 307 | 307 | 307 | 307 | 307 | 307 | 307 |
| Apaty | 307 | 307 | 307 | 307 | 306 | 307 | 307 | 307 | 307 | 307 | 307 | 307 |
| Disinhibition | 307 | 307 | 307 | 307 | 306 | 307 | 307 | 307 | 307 | 307 | 307 | 307 |
| Irritability | 307 | 307 | 308 | 307 | 306 | 307 | 307 | 307 | 308 | 307 | 308 | 308 |
| Aberrant motor behaviour | 307 | 307 | 307 | 307 | 306 | 307 | 307 | 307 | 307 | 307 | 307 | 307 |
| Night-time behaviour disturbances | 307 | 307 | 309 | 307 | 306 | 307 | 307 | 307 | 308 | 307 | 309 | 309 |
| Appetite and eating disturbances | 307 | 307 | 309 | 307 | 306 | 307 | 307 | 307 | 308 | 307 | 309 | 309 |

For the meta-analysis, we used n = 307 as the study sample size.

**2. APPROXIMATED CORRELATION MATRIX**

**Proitsi et al (2011)**

Sample size: 1850

|  | DEL | HAL | AGI | DEP | ANX | EUP | APA | DIS | IRR | AMB |
| --- | --- | --- | --- | --- | --- | --- | --- | --- | --- | --- |
| DEL | 1 | 0.588 | 0.403 | 0.211 | 0.243 | 0.177 | 0.205 | 0.323 | 0.372 | 0.387 |
| HAL | 0.588 | 1 | 0.303 | 0.159 | 0.183 | 0.134 | 0.244 | 0.243 | 0.28 | 0.291 |
| AGI | 0.403 | 0.303 | 1 | 0.236 | 0.271 | 0.186 | 0.339 | 0.452 | 0.615 | 0.405 |
| DEP | 0.211 | 0.159 | 0.236 | 1 | 0.336 | 0.112 | 0.204 | 0.195 | 0.218 | 0.143 |
| ANX | 0.243 | 0.183 | 0.271 | 0.336 | 1 | 0.128 | 0.234 | 0.224 | 0.251 | 0.28 |
| EUP | 0.177 | 0.134 | 0.186 | 0.112 | 0.128 | 1 | 0.046 | 0.297 | 0.172 | 0.205 |
| APA | 0.205 | 0.244 | 0.339 | 0.204 | 0.234 | 0.046 | 1 | 0.289 | 0.313 | 0.375 |
| DIS | 0.323 | 0.243 | 0.452 | 0.195 | 0.224 | 0.297 | 0.289 | 1 | 0.418 | 0.345 |
| IRR | 0.372 | 0.28 | 0.615 | 0.218 | 0.251 | 0.172 | 0.313 | 0.418 | 1 | 0.374 |
| AMB | 0.387 | 0.291 | 0.405 | 0.143 | 0.28 | 0.205 | 0.375 | 0.345 | 0.374 | 1 |

DEL, delusions; HAL, hallucinations, AGI, agitation; DEP, depression; ANX, anxiety; EUP, euphoria; APA, apathy; DIS, disinhibition; IRR, irritability; AMB, aberrant motor behaviour.

The approximated correlation matrix $\bar{R}$was computed from the CFA information in the Supplemental material (https://ars.els-cdn.com/content/image/1-s2.0-S0197458009001018-grsu1_lrg.jpg). Let $\Lambda$ be the matrix of factor loadings, $\Phi$ be the factor correlation matrix, and $\Theta$ be the matrix which contains error variances and covariances (Tabachnik & Fidell, 2014). Then,

$$\bar{R}= \Lambda\Phi\Lambda^{T}+ \Theta$$

$\Lambda$ =

| 0.884 | 0 | 0 | 0 |
| --- | --- | --- | --- |
| 0.665 | 0 | 0 | 0 |
| 0 | 0.541 | 0 | 0 |
| 0 | 0.621 | 0 | 0 |
| 0 | 0 | 0.816 | 0 |
| 0 | 0 | 0.754 | 0 |
| 0 | 0 | 0.382 | 0.232 |
| 0 | 0 | 0 | 0.307 |
| 0 | 0 | 0 | 0.560 |
| 0 | 0 | 0 | 0.669 |

$\Phi$ =

| 1 | 0.442 | 0.558 | 0.654 |
| --- | --- | --- | --- |
| 0.442 | 1 | 0.535 | 0.674 |
| 0.558 | 0.535 | 1 | 0.742 |
| 0.654 | 0.674 | 0.742 | 1 |

$\Theta$ =

| 0.287 | 0 | 0 | 0 | 0 | 0 | 0 | 0 | -0.119 | 0 |
| --- | --- | --- | --- | --- | --- | --- | --- | --- | --- |
| 0 | 0.558 | 0 | 0 | 0 | 0 | 0 | 0 | 0 | -0.101 |
| 0 | 0 | 0.707 | 0 | 0 | 0 | 0 | 0 | 0 | 0 |
| 0 | 0 | 0 | 0.615 | 0 | 0 | 0 | 0 | 0 | 0 |
| 0 | 0 | 0 | 0 | 0.335 | 0 | 0 | 0 | 0 | 0 |
| 0 | 0 | 0 | 0 | 0 | 0.431 | 0 | 0 | 0 | 0 |
| 0 | 0 | 0 | 0 | 0 | 0 | 0.669 | 0.139 | 0 | 0 |
| 0 | 0 | 0 | 0 | 0 | 0 | 0.139 | 0.905 | -0.126 | 0 |
| -0.119 | 0 | 0 | 0 | 0 | 0 | 0 | -0.126 | 0.686 | 0 |
| 0 | -0.101 | 0 | 0 | 0 | 0 | 0 | 0 | 0 | 0.553 |

All diagonal elements of $\bar{R}$ were then set to 1.0 before including the matrix in the meta-analysis.

Reference:

Tabachnick BG, Fidell LS. Principal Components and Factor Analysis. Using Multivariate Statistics. 6th ed. Essex: Pearson Education Limited; 2014.
