# Supplementary material for: Neuropsychiatric Inventory domains cluster into neuropsychiatric syndromes in Alzheimer's disease: A systematic review and meta‐analysis
Source: Brain Behav. 2022 Aug 8;12(9):e2734. doi: 10.1002/brb3.2734 (PMC9480932; doi:10.1002/brb3.2734)
Supplement: Supplementary file 4 — Supplemental Material 4: The information presented in this supplemental material concern the primary analysis of the NPI‐10 and its sensitivity analysis. [file BRB3-12-e2734-s005.docx]

**Supplemental material 4**

The information presented in this supplemental material concern the primary analysis of the NPI-10 and its sensitivity analysis.

**PRIMARY ANALYSIS (7 STUDIES; N = 5185 AD PATIENTS)**

**Supplemental material 4a: Pooled correlation matrix**

|  | DEL | HAL | AGI | DEP | ANX | EUP | APA | DIS | IRR | AMB |
| --- | --- | --- | --- | --- | --- | --- | --- | --- | --- | --- |
| DEL | 1 |  |  |  |  |  |  |  |  |  |
| HAL | 0.446303 | 1 |  |  |  |  |  |  |  |  |
| AGI | 0.34641 | 0.273168 | 1 |  |  |  |  |  |  |  |
| DEP | 0.222481 | 0.13655 | 0.230075 | 1 |  |  |  |  |  |  |
| ANX | 0.20324 | 0.113998 | 0.312803 | 0.414557 | 1 |  |  |  |  |  |
| EUP | 0.093493 | 0.102664 | 0.134617 | 0.10383 | 0.096567 | 1 |  |  |  |  |
| APA | 0.142397 | 0.13738 | 0.256063 | 0.26324 | 0.221031 | 0.049491 | 1 |  |  |  |
| DIS | 0.242045 | 0.171344 | 0.32088 | 0.170554 | 0.164797 | 0.279175 | 0.183911 | 1 |  |  |
| IRR | 0.286283 | 0.22239 | 0.563056 | 0.259658 | 0.289971 | 0.159324 | 0.246636 | 0.357279 | 1 |  |
| AMB | 0.241211 | 0.218855 | 0.312449 | 0.141864 | 0.23946 | 0.151527 | 0.258067 | 0.294136 | 0.30467 | 1 |

DEL, delusions; HAL, hallucinations, AGI, agitation; DEP, depression; ANX, anxiety; EUP, euphoria; APA, apathy; DIS, disinhibition; IRR, irritability; AMB, aberrant motor behaviour.

**Supplemental material 4b: Proportion of variability in NPI item correlations due to heterogeneity (I^2^)**

|  | DEL | HAL | AGI | DEP | ANX | EUP | APA | DIS | IRR |
| --- | --- | --- | --- | --- | --- | --- | --- | --- | --- |
| HAL | 0.8601 |  |  |  |  |  |  |  |  |
| AGI | 0.6414 | 0.6772 |  |  |  |  |  |  |  |
| DEP | 0.6181 | 0.6425 | Fixed at 0 |  |  |  |  |  |  |
| ANX | 0.4651 | 0.5218 | 0.8638 | 0.8611 |  |  |  |  |  |
| EUP | 0.4147 | 0.5694 | 0.1973 | 0.6672 | Fixed at 0 |  |  |  |  |
| APA | Fixed at 0 | 0.6396 | 0.3663 | 0.5715 | 0.7005 | 0.3051 |  |  |  |
| DIS | 0.4214 | Fixed at 0 | 0.6314 | Fixed at 0 | 0.2796 | Fixed at 0 | 0.5128 |  |  |
| IRR | 0.6398 | 0.5644 | 0.8525 | 0.4763 | 0.7837 | 0.1782 | 0.2674 | 0.7772 |  |
| AMB | 0.7901 | 0.1965 | 0.4839 | 0.0715 | 0.5958 | 0.0772 | 0.5732 | 0.609 | 0.3958 |

Some I^2^ are fixed at 0 because the between-study heterogeneity τ^2^ of an effect size was constrained to zero if its estimate reached the lower bound (1e-10) during pooling.

DEL, delusions; HAL, hallucinations, AGI, agitation; DEP, depression; ANX, anxiety; EUP, euphoria; APA, apathy; DIS, disinhibition; IRR, irritability; AMB, aberrant motor behaviour.

**Supplemental material 4c: Spalletta 2010 five factor model**

|  | Latent factors | | | | | Error variance |
| --- | --- | --- | --- | --- | --- | --- |
|  | F1 | F2 | F3 | F4 | F5 |  |
| **Indicator variables** |  |  |  |  |  |  |
| Delusions |  | 0.763 [0.7, 0.828] |  |  |  | 0.417 [0.315, 0.51] |
| Hallucinations |  | 0.587 [0.536, 0.639] |  |  |  | 0.655 [0.591, 0.713] |
| Agitation | 0.681 [0.645, 0.718] |  |  |  |  | 0.536 [0.485, 0.584] |
| Depression |  |  | 0.622 [0.561, 0.685] |  |  | 0.613 [0.532, 0.686] |
| Anxiety |  |  | 0.676 [0.61, 0.743] |  |  | 0.543 [0.448, 0.628] |
| Euphoria |  |  |  | 0.361 [0.325, 0.397] |  | 0.87 [0.842, 0.895] |
| Apathy |  |  |  |  | Fixed at 0.45 | Fixed at 0.80 |
| Disinhibition |  |  |  | 0.779 [0.724, 0.841] |  | 0.393 [0.292, 0.476] |
| Irritability | 0.674 [0.637, 0.711] |  |  |  |  | 0.546 [0.494, 0.595] |
| Aberrant motor behaviour | 0.51 [0.477, 0.544] |  |  |  |  | 0.74 [0.704, 0.773] |
| **Factor correlations** |  |  |  |  |  |  |
| F1 | 1 |  |  |  |  |  |
| F2 | 0.649 [0.586, 0.718] | 1 |  |  |  |  |
| F3 | 0.568 [0.507, 0.638] | 0.396 [0.332, 0.468] | 1 |  |  |  |
| F4 | 0.672 [0.612, 0.733] | 0.39 [0.336, 0.45] | 0.352 [0.298, 0.411] | 1 |  |  |
| F5 | 0.89 [0.802, 0.98] | 0.426 [0.345, 0.511] | 0.877 [0.747, 1.018] | 0.481 [0.371, 0.592] | 1 |  |

**Supplemental material 4d: Modified Spalletta 2010 four factor model**

|  | Latent factors | | | | Error variance |
| --- | --- | --- | --- | --- | --- |
|  | F1 | F2 | F3 | F4 |  |
| **Indicator variables** |  |  |  |  |  |
| Delusions |  | 0.759 [0.696, 0.822] |  |  | 0.424 [0.323, NA] |
| Hallucinations |  | 0.592 [0.54, 0.644] |  |  | 0.65 [0.585, 0.708] |
| Agitation | 0.679 [0.643, 0.716] |  |  |  | 0.538 [0.487, 0.586] |
| Depression |  |  | 0.525 [0.479, 0.573] |  | 0.724 [0.672, 0.771] |
| Anxiety |  |  | 0.599 [0.546, 0.653] |  | 0.641 [0.573, 0.702] |
| Euphoria |  |  |  | 0.361 [0.326, 0.397] | 0.87 [0.842, 0.894] |
| Apathy |  |  | 0.508 [0.465, 0.552] |  | 0.742 [0.695, 0.784] |
| Disinhibition |  |  |  | 0.778 [0.724, 0.84] | 0.394 [0.295, 0.476] |
| Irritability | 0.681 [0.644, 0.718] |  |  |  | 0.537 [0.484, 0.586] |
| Aberrant motor behaviour | 0.504 [0.471, 0.538] |  |  |  | 0.746 [0.711, 0.778] |
| **Factor correlations** |  |  |  |  |  |
| F1 | 1 |  |  |  |  |
| F2 | 0.651 [0.588, 0.721] | 1 |  |  |  |
| F3 | 0.708 [0.651, 0.768] | 0.415 [0.358, 0.477] | 1 |  |  |
| F4 | 0.674 [0.614, 0.735] | 0.391 [0.336, 0.45] | 0.414 [0.359, 0.473] | 1 |  |

Due to the small number of studies in the meta-analysis, we encountered convergence issues when estimating the upper 95% CI of the error variance of Delusions.

**SENSITIVITY ANALYSIS (6 STUDIES; N = 3335 AD PATIENTS)**

**Supplemental material 4e: Pooled correlation matrix**

|  | DEL | HAL | AGI | DEP | ANX | EUP | APA | DIS | IRR | AMB |
| --- | --- | --- | --- | --- | --- | --- | --- | --- | --- | --- |
| DEL | 1 |  |  |  |  |  |  |  |  |  |
| HAL | 0.410674 | 1 |  |  |  |  |  |  |  |  |
| AGI | 0.321033 | 0.251123 | 1 |  |  |  |  |  |  |  |
| DEP | 0.219635 | 0.124993 | 0.226793 | 1 |  |  |  |  |  |  |
| ANX | 0.186279 | 0.087965 | 0.313168 | 0.428491 | 1 |  |  |  |  |  |
| EUP | 0.060622 | 0.083314 | 0.110104 | 0.097516 | 0.079135 | 1 |  |  |  |  |
| APA | 0.107674 | 0.098059 | 0.221364 | 0.271946 | 0.212186 | 0.046288 | 1 |  |  |  |
| DIS | 0.20387 | 0.1316 | 0.278159 | 0.156998 | 0.138085 | 0.269291 | 0.14674 | 1 |  |  |
| IRR | 0.253969 | 0.194265 | 0.544053 | 0.266887 | 0.29078 | 0.149176 | 0.214965 | 0.331063 | 1 |  |
| AMB | 0.194779 | 0.181307 | 0.273993 | 0.140108 | 0.223324 | 0.124038 | 0.224844 | 0.265855 | 0.271348 | 1 |

DEL, delusions; HAL, hallucinations, AGI, agitation; DEP, depression; ANX, anxiety; EUP, euphoria; APA, apathy; DIS, disinhibition; IRR, irritability; AMB, aberrant motor behaviour.

**Supplemental material 4f:** **Proportion of variability in NPI item correlations due to heterogeneity (I^2^)**

|  | DEL | HAL | AGI | DEP | ANX | EUP | APA | DIS | IRR |
| --- | --- | --- | --- | --- | --- | --- | --- | --- | --- |
| HAL | 0.6889 |  |  |  |  |  |  |  |  |
| AGI | 0.6255 | 0.6548 |  |  |  |  |  |  |  |
| DEP | 0.5606 | 0.6468 | Fixed at 0 |  |  |  |  |  |  |
| ANX | 0.5347 | 0.517 | 0.8632 | 0.8405 |  |  |  |  |  |
| EUP | Fixed at 0 | 0.59 | 0.1644 | 0.7014 | Fixed at 0 |  |  |  |  |
| APA | Fixed at 0 | 0.4773 | Fixed at 0 | 0.0001 | 0.7697 | Fixed at 0 |  |  |  |
| DIS | 0.3258 | Fixed at 0 | Fixed at 0 | Fixed at 0 | 0.306 | Fixed at 0 | 0.1875 |  |  |
| IRR | 0.5611 | 0.534 | 0.8247 | 0.2757 | 0.7722 | 0.1652 | 0.0881 | 0.7397 |  |
| AMB | 0.6409 | Fixed at 0 | 0.1284 | Fixed at 0 | 0.6169 | Fixed at 0 | Fixed at 0 | 0.6314 | 0.2111 |

Some I^2^ are fixed at 0 because the between-study heterogeneity τ^2^ of an effect size was constrained to zero if its estimate reached the lower bound (1e-10) during pooling.

DEL, delusions; HAL, hallucinations, AGI, agitation; DEP, depression; ANX, anxiety; EUP, euphoria; APA, apathy; DIS, disinhibition; IRR, irritability; AMB, aberrant motor behaviour.

**Supplemental material 4g**: **Comparison of model fit indices across measurement models representing the NPI-10**

| Study and model | Factor 1 | Factor 2 | Factor 3 | Factor 4 | Factor 5 | Model χ^2^ | df | RMSEA [95% CI] | SRMR | TLI | CFI | AIC | BIC |
| --- | --- | --- | --- | --- | --- | --- | --- | --- | --- | --- | --- | --- | --- |
| Garre-Olmo 2011 Model 1 * | DEL  HAL  AMB | AGI  DEP  ANX  APA  IRR | EUP  DIS | - | - | 312.365 | 32 | 0.051 [0.046, 0.057] | 0.060 | 0.792 | 0.852 | 248.365 | 52.774 |
| Garre-Olmo 2011 Model 2 | DEL  HAL | AGI  DEP  ANX  APA  IRR | EUP  DIS  AMB | - | - | 302.660 | 32 | 0.050 [0.045, 0.056] | 0.055 | 0.799 | 0.857 | 238.660 | 43.068 |
| Garre-Olmo 2011 Model 3 | DEL  HAL | AGI  DEP  ANX  IRR | EUP  APA  DIS  AMB | - | - | 330.441 | 32 | 0.053 [0.048, 0.058] | 0.058 | 0.778 | 0.842 | 266.441 | 70.850 |
| Proitsi 2011 ** | EUP  APA  AMB | DEL  HAL | DEP  ANX | AGI  DIS  IRR | - | 309.860 | 29 | 0.054 [0.049, 0.059] | 0.048 | 0.770 | 0.852 | 251.860 | 74.606 |
| Vilalta-Franch 2010 follow-up model | AGI  DEP  ANX  IRR | DEL  HAL  APA | EUP  DIS  AMB | - | - | 398.578 | 32 | 0.059 [0.054, 0.064] | 0.068 | 0.727 | 0.806 | 334.578 | 138.987 |
| Connors 2018 *** | DEL  HAL | AGI  DEP  ANX  IRR | APA  DIS  AMB | - | - | 222.258 | 24 | 0.050 [0.044, 0.056] | 0.056 | 0.820 | 0.880 | 174.258 | 27.565 |
| Spalletta 2010 | AGI  IRR  AMB | DEL  HAL | DEP  ANX | EUP  DIS | APA | 110.510 | 26 | 0.031 [0.025, 0.037] | 0.035 | 0.923 | 0.955 | 58.510 | -100.408 |
| Modified Spalletta 2010 A | AGI  APA  IRR  AMB | DEL  HAL | DEP  ANX | EUP  DIS | - | 212.446 | 29 | 0.044 [0.038, 0.049] | 0.041 | 0.849 | 0.903 | 154.446 | -22.808 |
| Modified Spalletta 2010 B | AGI  IRR  AMB | DEL  HAL  APA | DEP  ANX | EUP  DIS | - | 256.276 | 29 | 0.049 [0.043, 0.05] | 0.057 | 0.814 | 0.880 | 198.276 | 21.021 |
| Modified Spalletta 2010 C | AGI  IRR  AMB | DEL  HAL | DEP  ANX  APA | EUP  DIS | - | 132.510 | 29 | 0.033 [0.027, 0.039] | 0.038 | 0.915 | 0.945 | 74.510 | -102.745 |
| Modified Spalletta 2010 D | AGI  IRR  AMB | DEL  HAL | DEP  ANX | EUP  APA  DIS | - | 290.107 | 29 | 0.052 [0.047, 0.058] | 0.050 | 0.786 | 0.862 | 232.107 | 54.852 |

* Same as the Vilalta-Franch 2010 baseline model; ** Disinhibition loaded only on F3; *** measurement model does not have euphoria. DEL, delusions; HAL, hallucinations, AGI, agitation; DEP, depression; ANX, anxiety; EUP, euphoria; APA, apathy; DIS, disinhibition; IRR, irritability; AMB, aberrant motor behaviour. CFI, comparative fit index; TLI, Tucker-Lewis index; RMSEA, root mean square error of approximation; SRMR, standardised root mean square residual; AIC, Akaike information criterion; BIC, Bayesian information criterion.

**Supplemental material 4h: Spalletta 2010 five factor model**

|  | Latent factors | | | | | Error variance |
| --- | --- | --- | --- | --- | --- | --- |
|  | F1 | F2 | F3 | F4 | F5 |  |
| **Indicator variables** |  |  |  |  |  |  |
| Delusions |  | 0.742 [0.676, 0.814] |  |  |  | 0.449 [0.337, 0.544] |
| Hallucinations |  | 0.558 [0.503, 0.615] |  |  |  | 0.688 [0.622, 0.747] |
| Agitation | 0.638 [0.597, 0.68] |  |  |  |  | 0.593 [0.538, 0.643] |
| Depression |  |  | 0.657 [0.588, 0.73] |  |  | 0.568 [0.467, 0.66] |
| Anxiety |  |  | 0.663 [0.592, 0.736] |  |  | 0.56 [0.458, 0.65] |
| Euphoria |  |  |  | 0.351 [0.304, 0.397] |  | 0.877 [0.842, 0.908] |
| Apathy |  |  |  |  | Fixed at 0.45 | Fixed at 0.80 |
| Disinhibition |  |  |  | 0.77 [0.7, 0.855] |  | 0.406 [0.387, 0.511] |
| Irritability | 0.662 [0.618, 0.707] |  |  |  |  | 0.562 [0.501, 0.619] |
| Aberrant motor behaviour | 0.478 [0.44, 0.517] |  |  |  |  | 0.771 [0.733, 0.807] |
| **Factor correlations** |  |  |  |  |  |  |
| F1 | 1 |  |  |  |  |  |
| F2 | 0.624 [0.554, 0.7] | 1 |  |  |  |  |
| F3 | 0.577 [0.505, 0.656] | 0.384 [0.309, 0.466] | 1 |  |  |  |
| F4 | 0.616 [0.543, 0.691] | 0.331 [0.269, 0.396] | 0.312 [0.248, 0.381] | 1 |  |  |
| F5 | 0.855 [0.761, 0.949] | 0.335 [0.236, 0.437] | 0.943 [0.818, 1.082] | 0.406 [0.298, 0.516] | 1 |  |

**Supplemental material 4i: Modified Spalletta 2010 four factor model**

|  | Latent factors | | | | Error variance |
| --- | --- | --- | --- | --- | --- |
|  | F1 | F2 | F3 | F4 |  |
| **Indicator variables** |  |  |  |  |  |
| Delusions |  | 0.734 [0.668, 0.805] |  |  | 0.461 [0.352, 0.554] |
| Hallucinations |  | 0.565 [0.51, 0.622] |  |  | 0.681 [0.613, 0.74] |
| Agitation | 0.641 [0.6, 0.682] |  |  |  | 0.59 [0.534, 0.64] |
| Depression |  |  | 0.566 [0.521, 0.612] |  | 0.68 [0.625, 0.728] |
| Anxiety |  |  | 0.626 [0.561, 0.692] |  | 0.608 [0.52, 0.685] |
| Euphoria |  |  |  | 0.352 [0.306, 0.398] | 0.876 [0.841, 0.907] |
| Apathy |  |  | 0.513 [0.47, 0.557] |  | 0.737 [0.689, 0.779] |
| Disinhibition |  |  |  | 0.768 [0.698, 0.837] | 0.41 [0.355, 0.513] |
| Irritability | 0.665 [0.621, 0.71] |  |  |  | 0.558 [0.496, 0.615] |
| Aberrant motor behaviour | 0.475 [0.437, 0.514] |  |  |  | 0.774 [0.736, 0.809] |
| **Factor correlations** |  |  |  |  |  |
| F1 | 1 |  |  |  |  |
| F2 | 0.629 [0.558, 0.704] | 1 |  |  |  |
| F3 | 0.696 [0.639, 0.756] | 0.36 [0.296, 0.429] | 1 |  |  |
| F4 | 0.618 [0.544, 0.692] | 0.332 [0.27, 0.398] | 0.351 [0.288, 0.416] | 1 |  |
