# Supplementary material for: Neuropsychiatric Inventory domains cluster into neuropsychiatric syndromes in Alzheimer's disease: A systematic review and meta‐analysis
Source: Brain Behav. 2022 Aug 8;12(9):e2734. doi: 10.1002/brb3.2734 (PMC9480932; doi:10.1002/brb3.2734)
Supplement: Supplementary file 5 — Supplemental Material 5: The information presented in this supplemental material concern the primary analysis of the NPI‐10 and its sensitivity analysis. [file BRB3-12-e2734-s003.docx]

Supplemental material 5

The information presented in this supplemental material concern the primary analysis of the NPI-10 and its sensitivity analysis.

**SECONDARY ANALYSIS (4 STUDIES; N = 2397 AD PATIENTS)**

**Supplemental material 5a: Pooled correlation matrix**

|  | DEL | HAL | AGI | DEP | ANX | EUP | APA | DIS | IRR | AMB | NBD | APP |
| --- | --- | --- | --- | --- | --- | --- | --- | --- | --- | --- | --- | --- |
| DEL | 1 |  |  |  |  |  |  |  |  |  |  |  |
| HAL | 0.373779 | 1 |  |  |  |  |  |  |  |  |  |  |
| AGI | 0.283836 | 0.254189 | 1 |  |  |  |  |  |  |  |  |  |
| DEP | 0.23437 | 0.077365 | 0.201951 | 1 |  |  |  |  |  |  |  |  |
| ANX | 0.166415 | 0.06143 | 0.291176 | 0.45158 | 1 |  |  |  |  |  |  |  |
| EUP | 0.061096 | 0.108612 | 0.096452 | 0.061129 | 0.074778 | 1 |  |  |  |  |  |  |
| APA | 0.088556 | 0.08303 | 0.190286 | 0.261479 | 0.23115 | 0.055569 | 1 |  |  |  |  |  |
| DIS | 0.201307 | 0.126751 | 0.272608 | 0.144389 | 0.146471 | 0.252308 | 0.159651 | 1 |  |  |  |  |
| IRR | 0.20968 | 0.176615 | 0.525455 | 0.227685 | 0.251417 | 0.142414 | 0.183099 | 0.327235 | 1 |  |  |  |
| AMB | 0.203975 | 0.15487 | 0.240573 | 0.118252 | 0.19719 | 0.104015 | 0.208304 | 0.246693 | 0.228959 | 1 |  |  |
| NBD | 0.177792 | 0.214422 | 0.199351 | 0.139579 | 0.175072 | 0.08951 | 0.156194 | 0.116298 | 0.155143 | 0.175964 | 1 |  |
| APP | 0.143845 | 0.093278 | 0.177512 | 0.187713 | 0.139551 | 0.07087 | 0.195263 | 0.164242 | 0.125023 | 0.157813 | 0.175363 | 1 |

DEL, delusions; HAL, hallucinations, AGI, agitation; DEP, depression; ANX, anxiety; EUP, euphoria; APA, apathy; DIS, disinhibition; IRR, irritability; AMB, aberrant motor behaviour; NBD, night-time behavioural disturbances; APP, appetite and eating abnormalities.

**Supplemental material 5b:** **Proportion of variability in NPI item correlations due to heterogeneity (I^2^)**

|  | DEL | HAL | AGI | DEP | ANX | EUP | APA | DIS | IRR | AMB | NBD |
| --- | --- | --- | --- | --- | --- | --- | --- | --- | --- | --- | --- |
| HAL | 0.3757 |  |  |  |  |  |  |  |  |  |  |
| AGI | 0 | 0.7214 |  |  |  |  |  |  |  |  |  |
| DEP | 0 | 0 | 0 |  |  |  |  |  |  |  |  |
| ANX | 0.5148 | 0 | 0.11 | 0.8681 |  |  |  |  |  |  |  |
| EUP | 0 | 0 | 0.0922 | 0.2739 | 0 |  |  |  |  |  |  |
| APA | 0 | 0.1718 | 0 | 0 | 0 | 0 |  |  |  |  |  |
| DIS | 0.2761 | 0 | 0 | 0 | 0.0451 | 0 | 0 |  |  |  |  |
| IRR | 0 | 0.7286 | 0.849 | 0 | 0 | 0.2759 | 0 | 0.7861 |  |  |  |
| AMB | 0.2596 | 0 | 0 | 0 | 0.568 | 0 | 0.0648 | 0.7065 | 0 |  |  |
| NBD | 0.5155 | 0 | 0 | 0 | 0.6246 | 0 | 0.6658 | 0 | 0 | 0.6134 |  |
| APP | 0 | 0 | 0.363 | 0 | 0 | 0 | 0 | 0 | 0 | 0.1881 | 0 |

Some I^2^ are fixed at 0 because the between-study heterogeneity τ^2^ of an effect size was constrained to zero if its estimate reached the lower bound (1e-10) during pooling.

**Supplemental material 5c: Kang 2010 four factor model**

|  | Latent factors | | | | Error variance |
| --- | --- | --- | --- | --- | --- |
|  | F1 | F2 | F3 | F4 |  |
| **Indicator variables** |  |  |  |  |  |
| Delusions |  |  | 0.749 [0.677, 0.833] |  | 0.439 [0.323, 0.542] |
| Hallucinations |  |  | 0.508 [0.445, 0.571] |  | 0.742 [0.674, 0.802] |
| Agitation | 0.693 [0.641, 0.747] |  |  |  | 0.52 [0.443, 0.589] |
| Depression |  | 0.697 [0.616, 0.776] |  |  | 0.514 [0.397, 0.621] |
| Anxiety |  | 0.67 [0.59, 0.747] |  |  | 0.552 [0.442, 0.651] |
| Apathy |  |  |  | 0.497 [0.443, 0.553] | 0.753 [0.694, 0.803] |
| Disinhibition | 0.438 [0.391, 0.486] |  |  |  | 0.808 [0.764, 0.847] |
| Irritability | 0.632 [0.576, 0.69] |  |  |  | 0.6 [0.523, 0.669] |
| Night time behavioural disturbances |  |  |  | 0.431 [0.374, 0.49] | 0.814 [0.76, 0.86] |
| Appetite and eating abnormalities |  |  |  | 0.404 [0.355, 0.453] | 0.837 [0.795, 0.874] |
| **Factor correlations** |  |  |  |  |  |
| F1 | 1 |  |  |  |  |
| F2 | 0.542 [0.468, 0.63] | 1 |  |  |  |
| F3 | 0.576 [0.496, 0.659] | 0.415 [0.342, 0.497] | 1 |  |  |
| F4 | 0.637 [0.558, 0.721] | 0.686 [0.596, 0.794] | 0.443 [0.352, 0.537] | 1 |  |

Measurement model does not include Euphoria and Aberrant motor behaviour.
